# Single nucleotide polymorphisms and sickle cell disease-related pain: a systematic review

**DOI:** 10.3389/fpain.2023.1223309

**Published:** 2023-09-14

**Authors:** Gina M. Gehling, Keesha Powell-Roach, Diana J. Wilkie, Jennifer R. Dungan

**Affiliations:** ^1^ College of Nursing, University of Florida, Gainesville, FL, United States; ^2^ College of Nursing, Department of Community and Population Health, University of Tennessee Health Science Center, Memphis, TN, United States

**Keywords:** genetic variant, single nucleotide polymorphism, sickle cell disease, acute pain, chronic pain

## Abstract

**Background:**

Scientists have speculated genetic variants may contribute to an individual's unique pain experience. Although research exists regarding the relationship between single nucleotide polymorphisms and sickle cell disease-related pain, this literature has not been synthesized to help inform future precision health research for sickle cell disease-related pain. Our primary aim of this systematic review was to synthesize the current state of scientific literature regarding single nucleotide polymorphisms and their association with sickle cell disease-related pain.

**Methods:**

Using the Prisma guidelines, we conducted our search between December 2021–April 2022. We searched PubMed, Web of Science, CINAHL, and Embase databases (1998–2022) and selected all peer-reviewed articles that included reports of associations between single nucleotide polymorphisms and sickle cell disease-related pain outcomes.

**Results:**

Our search yielded 215 articles, 80 of which were duplicates, and after two reviewers (GG, JD) independently screened the 135 non-duplicate articles, we retained 22 articles that met the study criteria. The synthesis of internationally generated evidence revealed that this scientific area remains predominantly exploratory in nature, with only three studies reporting sufficient power for genetic association. Sampling varied across studies with a range of children to older adults with SCD. All of the included articles (*n* = 22) examined acute pain, while only nine of those studies also examined chronic pain.

**Conclusion:**

Currently, the evidence implicating genetic variation contributing to acute and chronic sickle cell disease-related pain is characterized by modestly powered candidate-gene studies using rigorous SCD-pain outcomes. Effect sizes and directions vary across studies and are valuable for informing the design of future studies. Further research is needed to replicate these associations and extend findings with hypothesis-driven research to inform precision health research.

## 1. Introduction

Sickle Cell Disease (SCD) is the most common single-gene hemoglobinopathy affecting around 6 million people globally, with an estimated 100,000 living in the United States (U.S.) (1). Pain is a hallmark of SCD pathophysiology, with recent studies demonstrating that adults with SCD suffer from multidimensional pain related to recurrent episodes of acute pain, commonly referred to as vaso-occlusive crisis (VOC), as well as ongoing chronic pain (2, 3). Pain as a result of VOC is the leading complication associated with SCD. VOCs have a sudden onset and occur as a result of polymerization of abnormal sickle hemoglobin, inflammation, and adhesion (4). VOC events are the most frequent cause of hospitalization and one of the most common predictors of death among people with SCD (5). Other sources of pain in SCD result from secondary disease-related pathophysiology such as splenic sequestration, gall bladder issues, necrosis of femur, ulcers, and more. There is evidence to support that mismanaged pain leads to inadequate pain control (6), an increase in mortality (7), and the need for health care utilization resulting in billions of dollars spent annually for people with SCD (6, 8).

Precision biomarker solutions for the management of SCD pain may reduce complications that lead to the excess morbidity, hospitalization, and mortality among this vulnerable population. Yet the underlying genetic contributions to SCD-related pain remain largely unknown. Evidence exists supporting a genetic link between an individual's unique experience with pain and the presence of SNPs (9). Moreover, scientists have discovered that a person's genetic makeup can play a critical role in pain medication metabolism and effectiveness (10). Yet, no current systematic review exists that characterizes the candidate genes associated with SCD-related pain pheno- and sub-phenotypes. Heritability estimates for chronic pain phenotypes indicate that genetic variation contributes to the presence and experience of pain (any chronic pain, $h_{\mathrm{adj}}^{2}$ = .16 ± .07, *p* = 0.02, severe chronic pain $h_{\mathrm{adj}}^{2}$ = .30 ± .13, *p* = 0.007) (11).

While heritability estimates are lacking specifically for SCD pain phenotypes, significant candidate gene associations for pain phenotypes in SCD patients have been reported. Given the large variability in pain among people with SCD, others have hypothesized genetic contribution to pain perception in SCD, as tested by genetic association studies in the literature (12, 13). Although research exists regarding the relationship between single nucleotide polymorphisms (SNPs) and SCD-related pain, the quality and strength of this literature has not been synthesized to describe key scientific gaps and to help inform future precision health research for SCD-related pain. Since pain is a highly complex and variable subjective phenotype, understanding the state-of-the-science regarding the SNPs and specific phenotypes and sub-phenotypes that have been studied will be useful to guide future precision biomarker research and clinical applications.

The purpose of this article is to report a systematic review of the candidate gene SNP association studies among patients with SCD-related pain phenotypes. Specifically, across the series of studies identified, we sought to characterize the state of the science in this field in terms of study designs, population characteristics, measures of the SCD-pain phenotypes, candidate genes and specific SNPs studied, associations between SNPs and the SCD-pain phenotypes, and an evaluation of strength of evidence. Synthesis of this body of literature will be useful to guide future research and for implementation of the knowledge in clinical settings to control the pain of SCD.

## 2. Methods

### 2.1. Search strategy

We conducted our search for articles published between December 2021–April 2022. We searched PubMed, Web of Science, CINAHL, and Embase databases (1998–2022) and selected all peer-reviewed articles that reported on associations between SNPs and SCD-related pain. Our search strategy included the following search terms: “sickle cell disease” OR “SCD” OR “beta-plus thalassemia” OR “beta-zero thalassemia” OR “hemoglobin-c” OR “sickle cell anemia” OR “sickle cell anaemia” OR “hemoglobinopathy” AND “pain character*” OR “pain phenotype” OR “pain*” OR “pain expression” OR “pain experience” OR “pain cris*” (iterations related to crisis) AND “genetic varia*” (iterations related to variation) OR “genetic mutation” OR “single nucleotide*” OR “SNP.” The complete search strategy appears in Supplementary Data S1. Because there is limited research on this topic, and because we wanted to understand the full scope of this research area, we decided not to limit the number of years included in the study. However, we expected that a majority of articles containing SNP associations would be published after 2003, when the Human Genome Project was completed, and the nature of this research expanded rapidly.

All articles from the search phase were then exported into a Prisma-guided literature review management software that facilitates selection and extraction among multiple team members (Covidence systematic review software, Veritas Health Innovation, Melbourne, Australia, 2022).

### 2.2. Selection criteria

**Figure 1:**
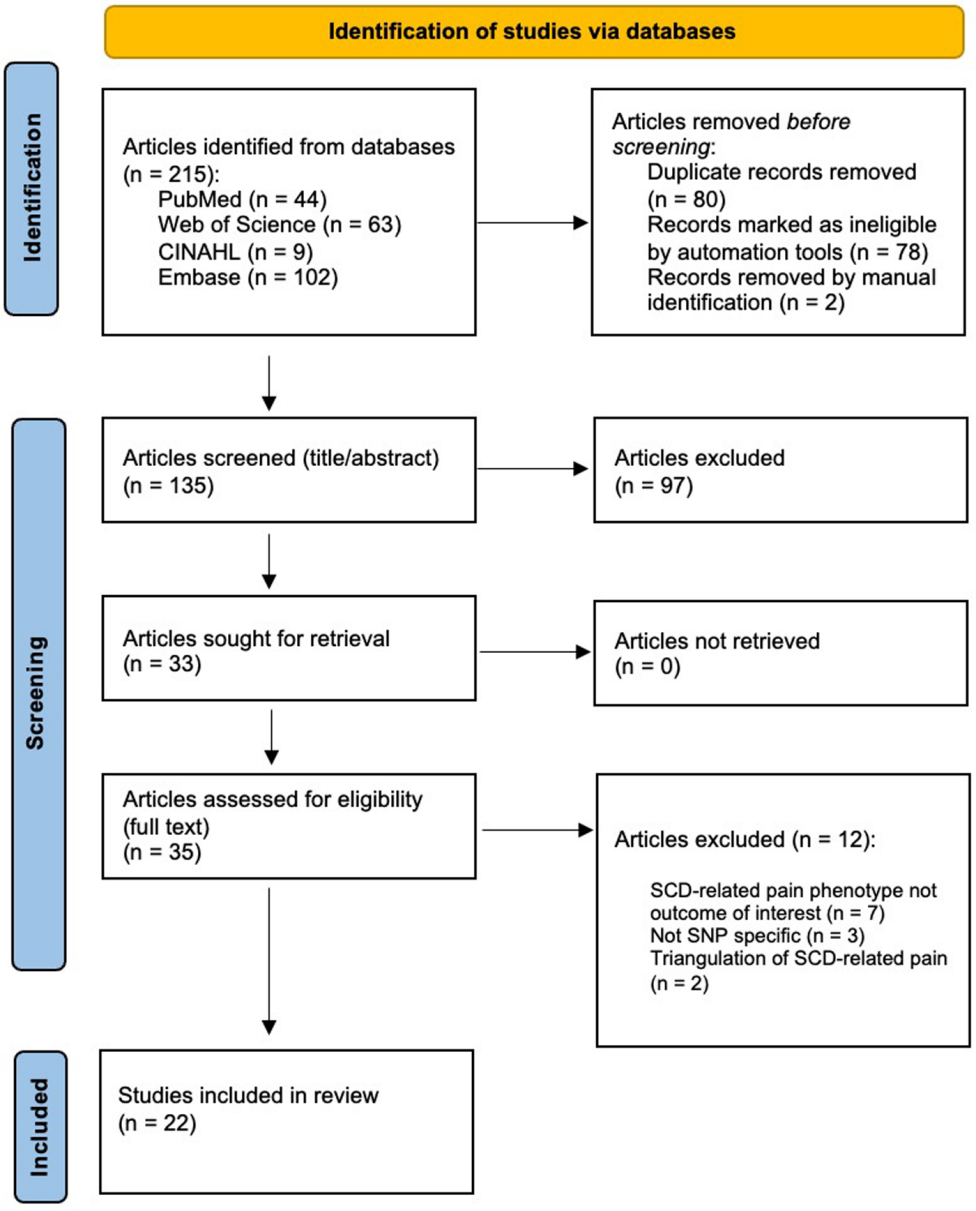
PRISMA diagram.

We selected articles based on criteria established by the team before initiating the screening. Inclusion criteria were as follows: (1) must include human participants with a diagnosis of SCD, which is inclusive of sickle cell anemia, (2) full article available in English, (3) focused on the association of SNPs or genetic variants and SCD-related pain phenotypes, and (4) published in a peer-reviewed journal. We included sickle cell crisis or vaso-occlusive crisis-focused articles if they specifically acknowledged a pain-related component (e.g., pain or painful crisis, pain event). Articles were excluded if they were: (1) literature reviews, (2) SNPs or genetic variants focused on SCD-related complications not specific to pain phenotype (e.g., acute chest syndrome, stroke), (3) full article unavailable, (4) animal study or model, (5) conference or meeting abstracts, or (6) there was a triangulation focus to a pain phenotype (e.g., SNPs with evidence supporting an association with fetal hemoglobin in heterogeneity of pain severity). Two authors (GG and JD) independently screened all articles and met to resolve any conflicts. After the title and abstract screening process, the two reviewers’ decisions to include or exclude an article had an inter-rater reliability proportionate agreement of 90.2% (Cohen's kappa of 0.78). During the full-text screening, the two investigators achieved a proportionate agreement of 84.4% (Cohen's kappa of 0.63). Figure 1 shows the PRISMA diagram indicating the flow of the article selection process. From the 215 identified articles, 22 articles met the inclusion criteria for further analysis.

### 2.3. Data extraction

GG was responsible for data extraction from the 22 articles that met study criteria into a literature matrix that KPR, DW, and JD independently reviewed for accuracy. Data extracted from each study appears in our literature matrix which can be found in Supplementary Data S2. Within our matrix are select sample characteristics (i.e., geographic location, age, gender, sickle cell genotype), SNPs and pain phenotype of interest, measures used to extract and sequence DNA, select significant statistical results, and a brief summary of significant study findings.

## 3. Results

### 3.1. Study characteristics

Of the 22 studies, 13 studies were conducted exclusively in the U.S., and one exclusively in the United Kingdom. Three multi-center international studies were conducted between the U.S. and another foreign country (*n* = 2 in the UK, *n* = 1 in Brazil), two in Africa (*n* = 1 Cameroon, *n* = 1 Tunisia), two in the Middle East (Bahrain), and one in India (Jabalpur). Very few investigators (*n* = 4) reported performing a power analysis, revealing majority of these studies were exploratory in nature (*n* = 18). Of those, three of the four studies reported sufficient power (79%–84%) to detect associations between SNPs of interest and an SCD-related pain phenotype (13–16).

### 3.2. Population characteristics

Sample sizes ranged from 107 to 1,275. Five studies sampled only children (14–18) under the age of 18 with a median sample size of *n* = 771. Four studies sampled only adults (19, 20–22) aged 18–70 years with a median sample size of *n* = 200. Thirteen studies sampled both children and adults (23–35) with a median sample size of *n* = 134. Of the 21 articles providing sufficient age data, we calculated a mean age of 24.8 ± 11.1 years (range 5–70) for most of the participants included in our review. There was a higher proportion of female participants than male participants (female 55.88% vs. male 44.12%).

### 3.3. Measures of SCD-related pain phenotypes

Pain phenotype characterization was most notably addressed within the pain measures. Since pain is subjective, self-report is considered the Gold Standard and most accurate measure of pain. Pain proxies are considered measures of pain which do not involve the patient's self-report, such as medical record review.

#### 3.3.1. Acute SCD pain phenotype

We found consistency among articles in phenotyping SCD-related pain through the use of proxies. All 22 studies used some form of health care utilization as a proxy for acute pain in SCD. Ten studies used acute health care utilization as a result of pain crisis obtained through medical record review and biweekly telephone calls (19, 24–29, 31–33). Other researchers used acute care utilization as a surrogate for acute pain defined by any visits to an acute care center, emergency center, or hospitalization. For example, five studies used vaso-occlusive crisis or pain crisis as a surrogate marker for acute pain (14, 20–23). Six studies used characteristics of hospitalization or provider intervention (i.e., utilization events) and pain management (e.g., required or did not require pharmaceutical intervention) as part of their proposed definition for pain (15, 18, 22, 23, 30, 35).

#### 3.3.2. Chronic SCD pain phenotype

Nine studies used self-reported pain at a routine clinic visit as a measure for chronic pain (24–29, 31–33). In these studies, investigators used the Composite Pain Index (CPI) score obtained from PAINReportIt as a self-report measure of the multidimensional pain experience (location, intensity, quality, pattern). Notably, CPI has been previously shown to be both a valid and reliable measure of chronic pain in SCD (36). In another study, investigators used the average pain intensity score (API) from PAINReportIt to identify an association between chronic pain heterogeneity and COMT SNPs (19).

### 3.4. Genetic associations with SCD-related pain

**Figure 2:**
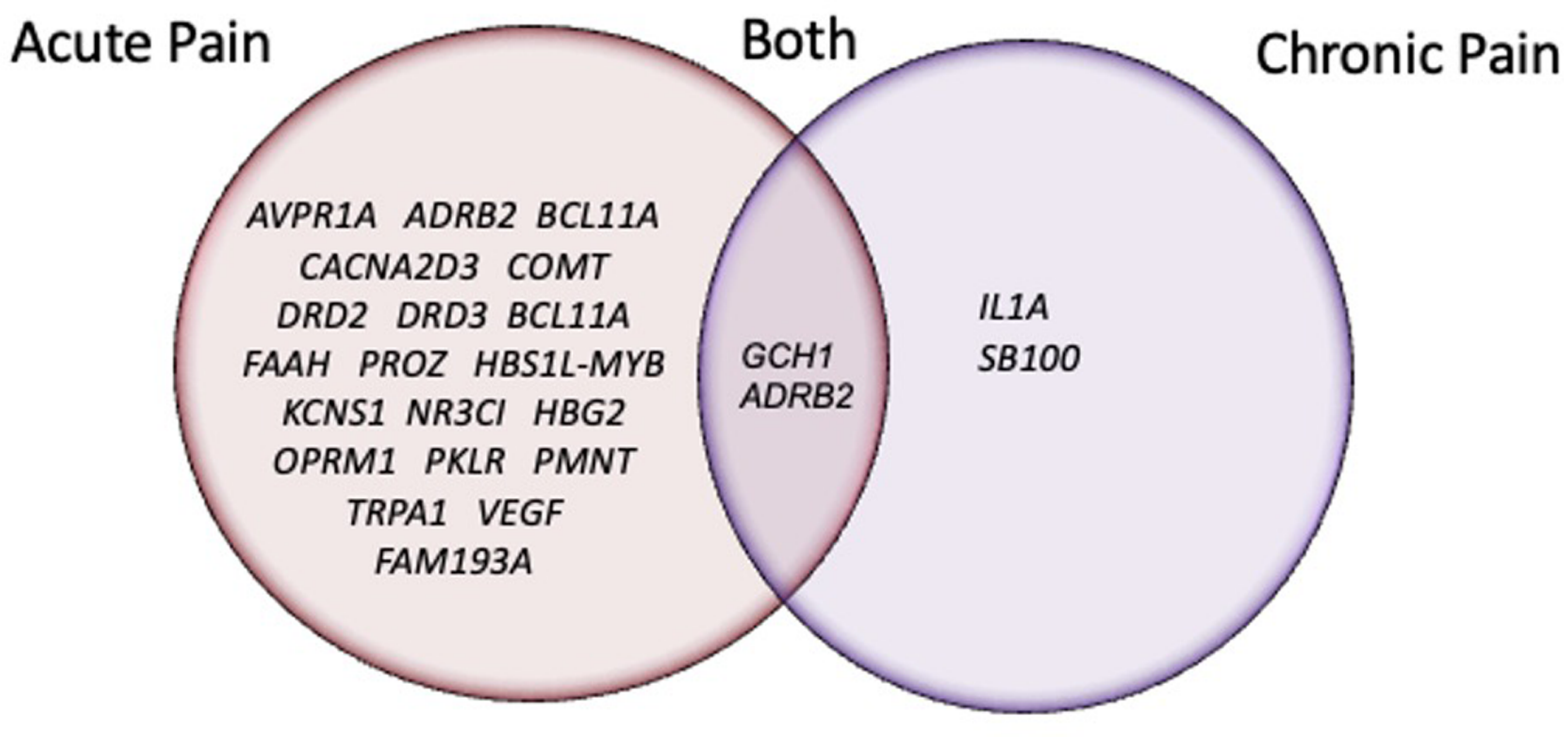
Venn-Diagram of genes with SNPs reported to have a significant association with an SCD pain-related phenotype.

Synthesis of the 22 articles revealed evidence of statistically suggestive associations between SCD-related acute pain heterogeneity in 20 gene regions with 48 respective SNPs (see Table 1) (14, 16, 20, 22, 23, 25, 26, 28, 29, 31–35). Additionally, there is evidence suggestive of a significant association between SCD-related chronic pain heterogeneity in 4 gene regions with 13 respective SNPs (see Table 2) (24, 27, 28, 33). Two genes, *ADRB2* (28) and *GCH1* (33) have preliminary evidence indicating they may play a role in variation among both acute and chronic pain in SCD. Figure 2 displays all genes reported to have significant associations with SCD pain phenotypes.

**Table 1:** Reported significant gene associations with SCD-related acute pain.

Gene	SNP	Citation
*ADRB2*
	rs1042711	(28)
	rs11168070	(35)
	rs11959427	
	rs1801704	
	rs1042713	
	rs17778257	
	rs12654778	
*AVPR1A*	rs10877969	(31, 35)
*BCL11A*	rs11886868	(35)
	rs4671393	
*CACNA2D3*	rs6777055	(35)
*COMT*	Val158Met/rs4680	(20, 25, 35)
	rs4633	(20)
	rs165599	
	rs6269	(35)
*DRD2*	rs4274224	(35)
*DRD3*	rs4680	(25)
*FAAH*	rs4141964	(35)
*FAM193A*	rs11732673	(23)
*GCH1*	rs3783641	(33)
	rs8007267	(22, 33)
	rs2878172	(22)
	rs7147286	
*HBG2*	rs7482144	(35)
*HBS1l-MYB*	rs28384513	(35)
	rs9494142	
*KCNS1*	rs734784	(35)
*OPRM1*	rs1799971	(35)
*PKLR*	rs2071053	(34)
* *	rs8177970	
	rs116244351	
	rs114455416	
	rs12741350	
	rs3020781	
	rs8177964	
*PMNT*	rs2934965	(32)
	rs876493	
	rs2941523	
*PROZ*	rs3024731	(17)
	rs3024735	
*NR3CI*	rs33389	(29)
	rs2963155	
	rs9324918	
*TRPA1*	rs920829	(26)
*VEGF*	rs2010963	(15)
	rs8333068	
	rs3025020	

**Table 2:** Reported significant gene associations with SCD-related chronic pain.

Gene	SNP	Citation
*ADRB2*	rs17778257	(28)
	rs12654778	
	rs11168070	
	rs11959427	
	rs1042711	
	rs1801704	
	rs1042713	
*GCH1*	rs8007267	(33)
*IL1A*	rs1800587	(24)
*SB100*	rs9722	(27)
	rs1051169	
	rs9983698	
	rs11911834	

Moreover, we compiled a summary of magnitude and direction of effect for candidate genes identified as having a possible association with SCD-related pain during our review (see Table 3). As an additional point of reference, we have provided a summary gene annotation table which includes information on gene function for candidate genes identified during our review (Supplementary Data S3).

**Table 3:** Summary table reporting magnitude of effect for single SNPs with reported evidence suggestive of association with SCD-related pain phenotypes.

Acute Pain Phenotype: ↑ Effect			Chronic Pain Phenotype: ↑ Effect		
*BCL11A*	rs11886868 effect −0.155, SE 0.17	(35)	*IL1A*	rs1800587 *β* (add) 3.85, 95% CI, 0.15–7.56	(24)
	rs4671393 effect −0.33, SE 0.13			rs1800587 β (dom) 5.66, 95% CI, −1.26–12.58	
	rs4671393 effect −0.23, SE 0.09			rs1800587 β (rec) 4.62, 95% CI, −0.77–10.02	
*CACNA2D3*	rs6777055 effect −0.17, SE 0.07	(35)	*ADRB2* (↑ *CPI*)	rs1042711 β (add) 10.86, 95% CI, 4.85–16.86	(28)
	rs6777055 effect −0.18, SE 0.07			rs1042711 β (dom) 11.28, 95% CI, 4.74–17.81	
				rs11168070 β (add) 5.99, 95% CI, 1.12–10.85	
				rs11168070 β (dom) 5.67, 95% CI, 0.58–10.76	
				rs11959427 β (add) 5.69, 95% CI, 0.82–10.56	
				rs11959427 β (dom) 5.34, 95% CI, 0.23–10.46	
				rs1801704 β (add) 5.26, 95% CI, 0.49–10.02	
*COMT*	rs4680 OR 0.32, 95% CI, 0.12–0.83	(25)	*ADRB2* (↓ CPI)	rs1042713 β (add) −5.73, 95% CI, −9.24–−2.23	(28)
				rs1042713 β (rec) −8.53, 95% CI, −14.33–−2.72	
				rs1042713 β (dom) −6.62, 95% CI, −12.38–−0.86	
				rs12654778 β (add) −4.52, 95% CI, −8.28–−0.75	
				rs12654778 β (dom) −5.87, 95% CI, −10.54–−1.20	
				rs17778257 β (add) −4.39, 95% CI, −8.40–−0.38	
				rs17778257 β (dom) −5.98, 95% CI, −11.09–−0.87	
*COMT*	rs6269 effect 0.14, SE 0.04	(35)	*S100B*	r9983698 β (add) 4.19, 95% CI, 0.20–8.17	(27)
				r9983698 β (dom) 5.44, 95% CI, 0.53–10.34	
				rs9722 β (add) 5.24, 95% CI, 1.96–8.52	
				rs9722 β (dom) 8.08, 95% CI, 3.02–13.14	
*DRD2*	rs4274224 effect 0.15, SE 0.07	(35)			
*DRD3*	rs6280 OR 0.86, 95% CI, 1.09–5.12	(25)			
*FAAH*	rs4141964 effect −185, SE 0.04	(35)	** **		
*FAM193A*	CSSCD discovery	(23)	** **		
	rs11732673 β −0.34, SE 0.09				
	CSSCD replication				
	rs11732673β −0.37, SE 0.15				
*GCH1*	NIH cohort:	(22)	** **		
	rs8007267 OR (dom) 1.98, 95% CI, 1.11–3.53				
	rs2878172 OR (dom) 2.33, 95% CI, 1.23–4.41				
	rs7147286 OR (dom) 2.13, 95% CI, 1.21–3.78				
	CSSCD cohort:				
	rs8007267 OR (dom) 2.23, 95% CI, 1.29–3.84				
*GCH1*	rs3783641 IRR^a^ (add) 1.37, 95% CI, 1.05–1.81	(33)	** **		
	rs3783641 IRR^a^ (rec) 1.81, 95% CI, 1.11–3.05				
*KCNS1*	rs734784 effect −0.17, SE 0.05	(35)	** **		
	rs734784 effect −0.23, SE 0.07				
*NR3CI*	rs33389 IRR^b^ (add) 1.53, 95% CI, 1.09–2.15	(29)	** **		
	rs33389 IRR^b^ (rec) 1.64, 95% CI, 1.12–2.40				
	rs2963155 IRR^b^ (add) 1.80, 95% CI, 1.37–2.38				
	rs2963155 IRR^b^ (dom) 2.25, 95% CI, 1.13–4.49				
	rs2963155 IRR^b^ (rec) 2.07, 95% CI, 1.45–2.93				
	rs9324918 IRR^b^ (add) 1.43, 95% CI, 1.06–1.93				
*OPRM1*	rs1799971 effect −0.14, SE 0.04	(35)	** **		
*TRPA1*	rs920829 IRR^b^ (add) 1.44, 95% CI, 1.02–2.04	(26)	** **		
	rs920829 IRR^b^ (rec) 1.68, 95% CI, 1.15–2.48				
Acute Pain Phenotype: ↓ Effect			Chronic Pain Phenotype: ↓ Effect		
*HGB2*	rs7482144 effect −0.64, SE 0.18	(35)	*GCH1*	rs8007267 β (add) −3.76, 95% CI, −7.28–−0.24	(33)
*PMNT*	rs2934965 IRR^a^ (add) 0.61, 97.5% CI, 0.38–1.01	(32)		rs8007267 β (dom) −5.61, 95% CI, −10.37–−0.85	
	rs876493 IRR^a^ (add) 0.68, 97.5% CI, 0.51–0.93				
	rs876493 IRR^a^ (dom) 0.67, 97.5% CI, 0.47–0.97				
	rs2941523: IRR^a^ (add) 0.68, 97.5% CI, 0.47–0.99				
	rs2941523: IRR^a^ (rec) 0.12, 97.5% CI, 0.02–0.65				
*HBS1l-MYB*	rs9402686 effect −0.16, SE 0.58	(35)	*S100B*	rs1051169 β (add) −6.95, 95% CI, −10.33–−3.57	(27)
	rs4895441 effect −0.31, SE 0.14			rs1051169 β (add) −7.95, 95% CI, −12.82–−3.08	

OR, odds ratio; IRR^a^, incidence risk ratio; IRR^b^, incident rate ratio; β, Unstandardized regression coefficient; effect, Pseudo R-square or McFadden R-square (interpret effect with caution); SE, standard error.

## 4. Discussion

Prevalence of pain-related genes and associated variants found in individuals with SCD was previously reported by Jhun et al. (14). Yet, to our knowledge, no study has reported on the state of the science regarding the levels of evidence and study characteristics of associations between SNPs and pain phenotypes in SCD. We identified 22 articles across 2008–2022 reporting SNP associations with SCD-related pain. Statistically suggestive evidence associates SCD-related *acute pain* heterogeneity with 21 gene regions and 42 respective SNPs. Evidence for *chronic pain* gene associations is limited to reports of 2 gene regions with 5 respective SNPs. Moreover, 20 SNPs within 2 candidate genes had suggestive associations with both acute and chronic pain outcomes.

Acute health care utilization for pain management related to vaso-occlusive episodes or events were the most commonly tested acute pain phenotypes. Investigators obtained this information through patient or caretaker self-report and medical record review of acute care utilization for pain control or hospitalization rates. Since the high prevalence of SCD pain caused by sickling and VOC often leads to ineffective self-pain management and results in healthcare seeking behaviors, this method of measuring acute pain in SCD is widely accepted by SCD research experts. According to Ballas & Dampier, roughly 60% of individuals with SCD visit the hospital for VOC treatment at least once a year and close to 25% require hospital-based VOC treatment five or more times annually (6). Of note, Prestia and colleagues reported healthcare visits for active vaso-occlusive crisis account for 95% of hospitalizations with an average length of stay of 4.5 days (37).

An estimated 30%–40% of adolescents and adults with SCD live with chronic pain (38). Our synthesis on the state of the science revealed the chronic pain phenotype within this population varies widely. Some researchers have investigated the relationship between SNPs and chronic SCD pain using a composite pain index (CPI) from a well-validated tablet-based pain measurement tool, PAINReportIt (24–29, 31–33). Other investigators developed a chronic pain phenotype using average pain intensity scores (API) from PAINReportIt and a standard quantitative sensory testing protocol for heat, cold, and pressure thresholds, shown to be safe in those with SCD (39, 40). SCD pain is multidimensional in nature, with inconsistent frequency and severity, even in individuals with the same genotype. As such, the reviewed studies assumed the common disease-common variant hypothesis and studied *a-priori* candidate genes with biologic plausibility. For example, epinephrine, a neurotransmitter in pain signaling, has been shown to target the beta-2 adrenergic receptor (*ADRB2*) and polymorphisms in the *ADRB2* gene have been linked to chronic and acute pain in SCD, along with other conditions such as functional gastrointestinal and temporomandibular disorders (28, 35, 41, 42). Polymorphisms of the *GCH1* (GTP Cyclohydrolase 1) gene (22, 33), which encodes GTP cyclohydrolase enzyme and has been associated with pain sensitivity, has been shown to contribute to both chronic and acute pain by increasing or decreasing tetrahydrobiopterin (BH4) production, an essential enzyme cofactor responsible for the biosynthesis of neurotransmitters such as serotonin, dopamine, epinephrine, and norepinephrine.

Key gaps were identified in this synthesis. At this time, we could not find any studies exploring the genetic relationship between SNPs and neuropathic pain in SCD. In a quantitative sensory testing study on people with SCD who had previously diagnosed chronic pain, 32% of participants were identified as having allodynia, hyperalgesia, or both, all of which are widely accepted indicators of neuropathic pain among scientists in the pain research community (2). While there is preliminary evidence to support some individuals with SCD may experience neuropathic pain (NP), there is no objective measure for NP in this population. Providers primarily rely on the visual analog scale, pain descriptors and intensity (43–45). It is unclear if the NP observed in other conditions is equivalent to NP in SCD.

This study is not without its own limitations. A majority of the gene association studies were performed within the U.S., despite the predominance of SCD internationally. A lack of geographical representation hinders our ability to generalize our findings on a global scale. Smaller sample sizes and the potential for population-specific genetic effects are limitations of the reviewed work. Additionally, since much of the research conducted was exploratory in nature, definitive conclusions cannot be made at this time about the relationship between these candidate genes and SCD-related pain phenotypes. Even so, it is clear there is a need for future replication studies and hypothesis-driven work among individuals with SCD if we are to understand the genetic implications of SCD-related pain. Of note, we chose to focus specifically on human-derived candidate genes and acknowledge the value of experimental animal models and their relevance to future study in this area.

## 5. Conclusion

Without further investigation into SNPs and their association with SCD-related pain, morbidity and health care utilization for these individuals may remain high. Gene association research in SCD-related pain has been primarily focused on increasing our understanding of the role of genetic variants on acute pain in SCD. There is a clear need for replication and hypothesis-driven gene association studies. Moreover, we believe there is an essential need to investigate genetic associations within all subtypes of pain in the sickle cell population. Further research has the potential to inform precision health practices on how best to manage all pain phenotypes in SCD.
