# Supplementary material for: Single nucleotide polymorphisms and sickle cell disease-related pain: a systematic review
Source: Front Pain Res (Lausanne). 2023 Sep 14;4:1223309. doi: 10.3389/fpain.2023.1223309 (PMC10538969; doi:10.3389/fpain.2023.1223309)
Supplement: Supplementary file 1 [file Datasheet1.docx]

Supplemental Data 1. Search strategy, results, and article criteria for inclusion.

Question:

"Which single nucleotide polymorphisms in the existing scientific literature have been associated with pain phenotypes in people living with sickle cell disease?"

Terms:

("sickle cell disease" or "SCD" or "beta-plus thalassemia" or "beta-zero thalassemia" or "hemoglobinopathy" or "sickle cell anemia" or "sickle cell anaemia") AND ("pain" or "pain character*" or "pain phenotype" or "pain expression" or "pain experience" or "pain cris*) AND ("genetic varia*" or "genetic mutation" or "single nucleotide*" or "snp")

Databases:

Pubmed

((sickle cell disease[Title/Abstract] OR "SCD*"[Title/Abstract] OR "beta-plus thalassemia"[Title/Abstract] OR "beta-zero thalassemia"[Title/Abstract] OR "hemoglobin-c"[Title/Abstract] OR "sickle cell anemia"[Title/Abstract] OR sickle cell anaemia[Title/Abstract] OR "hemoglobinopathy"[Title/Abstract]) AND ("pain character*"[Title/Abstract] OR "pain phenotype"[Title/Abstract] OR "pain*"[Title/Abstract]OR "pain expression"[Title/Abstract] OR "pain experience"[Title/Abstract] OR "pain cris*"[Title/Abstract])) AND ("genetic varia*"[Title/Abstract] OR "genetic mutation"[Title/Abstract] OR "single nucleotide*"[Title/Abstract] OR "snp"[Title/Abstract]) 4/4/22 44 results

Web of Science:

ALL=(sickle cell disease OR "SCD*" OR "beta-plus thalassemia" OR "beta-zero thalassemia" OR "hemoglobin-c" OR "sickle cell anemia" OR sickle cell anaemia OR "hemoglobinopathy") AND ALL=("pain character*" OR "pain phenotype" OR "pain*" OR "pain expression" OR "pain experience" OR "pain cris*") AND ALL=("genetic varia*" OR "genetic mutation" OR "single nucleotide*" OR "snp")

4/4/22 – 63 results

CINAHL:

("sickle cell disease" or "scd*" or "beta-plus thalassemia" or "beta-zero thalassemia" or "hemoglobin-c" or "sickle cell anemia" or "sickle cell anaemia" or "sickle hemoglobinopathy") AND ("pain" or "pain character*" or "pain phenotype" or "pain expression" or "pain experience" or "pain cris*") AND ("genetic varia*" or "genetic mutation" or "single nucleotide*" or "snp") 4/4/22 – 9 results

Embase:

('sickle cell disease'/exp OR 'sickle cell disease' OR 'scd' OR 'beta-plus thalassemia' OR 'beta-zero thalassemia' OR 'hemoglobin-c'/exp OR 'hemoglobin-c' OR 'sickle cell anemia'/exp OR 'sickle cell anemia' OR 'sickle cell anaemia'/exp OR 'sickle cell anaemia' OR 'sickle hemoglobinopathy') AND ('pain'/exp OR 'pain' OR 'pain character*' OR 'pain phenotype' OR 'pain expression' OR 'pain experience' OR 'pain cris*') AND ('genetic varia*' OR 'genetic mutation' OR 'single nucleotide*' OR 'snp'/exp OR 'snp') AND ([article]/lim OR [data papers]/lim OR [review]/lim) AND [humans]/lim^

^Where lim = selected limits for human, publication type: article, data papers, review 4/4/22 – 102 results

Inclusion criteria:

1) human participants diagnosed with sickle cell disease or sickle cell anemia

2) full research article available in English

3) focused on association of SNPs or genetic variants and SCD-related pain phenotypes

a) sickle cell crisis or vaso-occlusive crisis included if acknowledged the pain component (e.g., painful sickle cell crisis, painful crisis, pain crisis)

b) chronic pain

c) neuropathic pain

4) published in peer-reviewed journal

Exclusion criteria:

1) literature reviews

2) single nucleotide polymorphism/genetic variants of SCD-related complications other than pain phenotype (e.g., acute chest syndrome, stroke)

3) full article unavailable

4) animal study/model

5) conference meetings or abstracts

6) triangulation to pain phenotype (not directly related to SCD-related pain)
