# Supplementary material for: Single nucleotide polymorphisms and sickle cell disease-related pain: a systematic review
Source: Front Pain Res (Lausanne). 2023 Sep 14;4:1223309. doi: 10.3389/fpain.2023.1223309 (PMC10538969; doi:10.3389/fpain.2023.1223309)
Supplement: Supplementary file 2 [file Datasheet2.docx]

| **SNPs and SCD Literature Matrix.** | | | | | | | |
| --- | --- | --- | --- | --- | --- | --- | --- |
| **(Citation)**  **Sample Characteristics** | **Pain Phenotype** | **dbSNP(s) ID** | | **Measures** | | **Results** | **Conclusion** |
| **(Hu et al/2016)^1^**  115 individuals with SCD (Illinois, US)  Age (mean, SD) in years:  34.1 ± 12.3  range 15-70  Sex (n, %):  female 78 (68)  male 37 (32)  SC Type (n, %):  SCD-SS: 93 (81)  SCD-SC: 11 (10)  SCD-β^+^: 5 (4)  SCD-β^ο^: 5 (4)  SCD-α: 1 (1)  Ethnicity (n, %):  African American 11 (97)  Hispanic 3 (3)  Caucasian 1 (1) | Acute  Chronic | Interleukin 1 Alpha (*IL1A*)   - rs1800587 | | Acute phenotype:  Acute care utilization over past 12 months (defined as the number of admissions to the emergency department and/or acute care center as a result of pain crisis from chart review and biweekly telephone calls) after completing baseline pain assessment.  Chronic phenotype:  Subjects completed a baseline pain assessment using PAIN*Report*It during a routine outpatient visit (i.e., not during pain crisis or other urgent care visits to the clinic). A composite score (CPI) was computed as a representation of the multidimensional pain experience (location, intensity, quality, pattern) at baseline.  DNA extraction:  (1) blood samples: modified salting out procedure or QuickGene-mini80 isolation device & QuickGene DNA whole blood extraction method (2) buccal samples: modified phenol/chloroform procedure  Genotype:  MassARRAY iPlex Platform, subsequently measured via MALDI-TOF MS | | Effects of *IL1A* (rs1800587) on chronic pain (B = unstandardized regression coefficient):  Additive: v=3.85 β (95% CI 0.15-7.56); **p=.042**  Recessive: β=4.62 (95% CI -.077-10.02); **p=.0092** | Findings from this exploratory study suggest evidence of an association between *IL1A* (rs1800587) and the heterogeneity of chronic pain in SCD. |
| **(Jhun et al., 2014)^2^**  130 individuals with SCD (Illinois, US)  Age (mean, SD) in years:  34.78 ± 11.35  range 19-70  Sex (n, %):  female 86 (66.2)  male 44 (33.8)  SC Type (n, %):  SCD-SS: 99 (76.2)  SCD-SC: 15 (11.5)  SCD-β^+^, SCD-β^ο^, SCD-α): 16 (12.3)  Ethnicity (n, %):  African American 127 (97.7)  Caucasian 1 (0.8)  Hispanic 2 (1.5) | Acute  Chronic | Catechol-O-Methyltransferase (*COMT*)   - Rs4680 (Val158Met)   Dopamine Receptor D3 (*DRD3*)   - Rs6280 (Ser9Gly) | | Acute phenotype:  Acute care utilization over past 12 months (defined as the number of admissions to the emergency department and/or acute care center as a result of pain crisis from chart review and biweekly telephone calls) after completing baseline pain assessment.  Chronic phenotype:  Subjects completed a baseline pain assessment using PAIN*Report*It during a routine outpatient visit (i.e., not during pain crisis or other urgent care visits to the clinic). A composite score (CPI) was computed as a representation of the multidimensional pain experience (location, intensity, quality, pattern) at baseline.  DNA extraction:  (1) blood samples: modified salting out procedure or QuickGene-mini80 isolation device & QuickGene DNA whole blood extraction method (2) buccal samples: modified phenol/chloroform procedure  Genotype:  Performed according to previously published methods for *COMT* Val158MET and DRD3 Ser9Gly | | Association of *DRD3* Ser9Gly on number of events with utilization:  Utilization, n(%):  0: heterozygotes 13 (76.5), homozygotes 4 (23.5),  ≥1: heterozygotes 52 (46.0), homozygotes 61 (54.0)  OR (95% CI): 3.81 (1.17, 12.41); **p=0.035** | Findings from this exploratory study suggest evidence of an association between *DRD3* (rs4680) and the heterogeneity of acute pain in SCD. |
| **(Jhun et al., 2018)^3^**  132 individuals with SCD (Illinois, US)  Age (mean, SD) in years:  34.2 ± 11.8  range 15-70  Sex (n, %):  female 86 (65.2)  male 46 (34.8)  SC Type (n, %):  SCD-SS: 102 (77.3)  SCD-SC: 15 (11.4)  Others (SCD-β^+^,  SCD-β^ο^, SCD-α): 15 (11.4) | Acute  Chronic | Transient Receptor Potential Cation Channel Subfamily V Member 1 (*TPRV1*)   - rs1947913 - rs13279503 - rs13255063 - rs1025928 - rs3735942 - rs3735943 - rs920829 - rs1443952   Transient Receptor Potential Cation Channel Subfamily A Member 1 (*TRPA1*)   - rs8065080 - rs224534 - rs222747 | | Acute phenotype:  Acute care utilization over past 12 months (defined as the number of admissions to the emergency department and/or acute care center as a result of pain crisis from chart review and biweekly telephone calls) after completing baseline pain assessment.  Chronic phenotype:  Subjects completed a baseline pain assessment using PAIN*Report*It during a routine outpatient visit (i.e., not during pain crisis or other urgent care visits to the clinic). A composite score (CPI) was computed as a representation of the multidimensional pain experience (location, intensity, quality, pattern) at baseline.  DNA extraction:  (1) blood samples: modified salting out procedure or QuickGene-mini80 isolation device & QuickGene DNA whole blood extraction method (2) buccal samples: modified phenol/chloroform procedure  Genotype:  MassARRAY iPlex Platform used for all SNPs except rs224534 and rs222747 (success rate >90%) | | Effects of *TRPA1* (rs920829) on utilization (IRR = incident rate ratio):  IRR [95% CI]   - rs920829   **Add: 1.44 [1.02-2.04] p=0.027**; Dom: 0.82 [0.35-2.11] p=0.656; **Rec: 1.68 [1.15-2.48] p=0.008** | Findings from this exploratory study suggest evidence of an association between *TRPA1* (rs920829) and the heterogeneity of acute pain in SCD. |
| **(Jhun et al., 2020)^4^**  136 individuals with SCD (Illinois, US)  Age (mean, SD) in years:  34.00 ± 11.7  range 15-70  Sex (n, %):  female 89 (65)  male 47 (35)  SC Type (n, %):  SCD-SS: 105 (77)  SCD-SC: 15 (11)  SCD-β^+^: 8 (6)  SCD-β^ο^: 7 (5)  SCD-α: 1 (1)  Ethnicity (n, %):  African American 132 (97)  Caucasian 1 (1)  Hispanic 3 (2) | Acute  Chronic | *S100* Calcium Binding Protein B (*S100B*)   - rs1051169 - rs11911834 - rs9983698 - rs9722 | | Acute phenotype:  Acute care utilization over past 12 months (defined as the number of admissions to the emergency department and/or acute care center as a result of pain crisis from chart review and biweekly telephone calls) after completing baseline pain assessment.  Chronic phenotype:  Subjects completed a baseline pain assessment using PAIN*Report*It during a routine outpatient visit (i.e., not during pain crisis or other urgent care visits to the clinic). A composite score (CPI) was computed as a representation of the multidimensional pain experience (location, intensity, quality, pattern) at baseline.  DNA extraction:  (1) blood samples: modified salting out procedure or QuickGene-mini80 isolation device & QuickGene DNA whole blood extraction method (2) buccal samples: modified phenol/chloroform procedure  Genotype:  MassARRAY iPlex Platform used for all SNPs except rs224534 and rs222747 (success rate >90%) | | Effect of *S100B* SNPs on acute (utilization) and chronic (CPI ) pain phenotypes in SCD (IRR = incident rate ratio, B = unstandardized regression coefficient):   - rs9722   CPI: B (95% CI)  Additive: 5.24 (1.96, 8.52) **adj.** **p=0.005**  Dominant: 8.08 (3.02, 13.14) **adj. p=0.005**   - rs1051169   CPI: B (95% CI)  Additive: -6.95 (-10.33, -3.57) **adj. p=0.001**  Dominant: -7.95 (-12.82, -3.08) **adj. p=0.005** | Findings from this exploratory study suggest evidence of an association between *S100B* (rs9722, rs1051169) in the heterogeneity of chronic pain in SCD. |
| **(Jhun et al., 2019)^5^**  115-136 individuals with SCD (Illinois, US)  Age (mean, SD) in years:  34.00 ± 11.7  range 15-70  Sex (n, %):  female 89 (65)  male 47 (35)  SC Type (n, %):  SCD-SS: 105 (77)  SCD-SC: 15 (11)  SCD-β^+^: 8 (6)  SCD-β^ο^: 7 (5)  SCD-α: 1 (1)  Ethnicity (n, %):  African American 132 (97)  Caucasian 1 (1)  Hispanic 3 (2) | Acute  Chronic | Beta-2 Adrenergic Receptor (*ADRB2*)   - rs11958940 - rs1432622 - rs17778257 - rs2895795 - rs2400707 - rs2053044 - rs12654778 - rs11168070 - rs11959427 - rs1042711 - rs1801704 - rs1042713 - rs1042717 - rs1042718 - rs1042719 - rs1042720 | | Acute phenotype:  Acute care utilization over past 12 months (defined as the number of admissions to the emergency department and/or acute care center as a result of pain crisis from chart review and biweekly telephone calls) after completing baseline pain assessment.  Chronic phenotype:  Subjects completed a baseline pain assessment using PAIN*Report*It during a routine outpatient visit (i.e., not during pain crisis or other urgent care visits to the clinic). A composite score (CPI) was computed as a representation of the multidimensional pain experience (location, intensity, quality, pattern) at baseline.  DNA extraction:  (1) blood samples: modified salting out procedure or QuickGene-mini80 isolation device & QuickGene DNA whole blood extraction method (2) buccal samples: modified phenol/chloroform procedure  Genotype:  MassARRAY iPlex Platform used for all SNPs except rs224534 and rs222747 (success rate >90%) | | Effect of *ADRB2* SNPs on chronic pain (CPI) (B = unstandardized regression coefficient):   - rs17778257: B (95% CI)   Additive: -4.39 (-8.40, -0.38) **p=0.03**  Dominant: -5.98 (-11.09, -0.87) **p=0.02**   - rs12654778: B (95% CI)   Additive: -4.52 (-8.28, -0.75) **p=0.02**  Dominant: -5.87 (-10.54, -1.20) **p=0.01**   - rs11168070: B (95% CI)   Additive: 5.99 (1.12, 10.85) **p=0.02**  Dominant: 5.67 (0.58, 10.76) **p=0.03**   - rs11959427: B (95% CI)   Additive: 5.69 (0.82, 10.56) **p=0.02**  Dominant: 5.34 (0.23, 10.46) **p=0.04**   - rs1042711: B (95% CI)   Additive: 10.86 (4.85, 16.86) **p=<0.001**  Dominant: 11.28 (4.74, 17.81) **p=0.001**   - rs1801704: B (95% CI)   Additive: 5.26 (0.49, 10.02) **p=0.03**   - rs1042713: B (95% CI)   Additive: -5.73 (-9.24, -2.23) **p=0.002**  Recessive: -8.53 (-14.33, -2.72) **p=0.004**  Dominant: -6.62 (-12.38, -0.86) **p=0.02** | Findings from this exploratory study suggest evidence of an association between *ADRB2* (rs1042711, rs11168070, rs11959427, rs1801704,  rs1042713, rs17778257, rs12654778) and the heterogeneity of chronic pain in SCD. |
| **(Jhun et al, 2018)^6^**  136 individuals with SCD (Illinois, US)  Age (mean, SD) in years:  34.00 ± 11.7  range 15-70  Sex (n, %):  female 89 (65.4)  male 47 (34.6)  SC Type (n, %):  SCD-SS: 105 (77.2)  SCD-SC: 15 (11.0)  SCD-β^+^: 8 (5.9)  SCD-β^ο^: 7 (5.1)  SCD-α: 1 (0.7)  Ethnicity (n, %):  African American 132 (97.1)  Caucasian 1 (0.7)  Hispanic 3 (2.2) | Acute  Chronic | Nuclear Receptor Subfamily 3 Group C Member 1 (*NR3C1*)   - rs33389 - rs2963155 - rs9324918 | | Acute phenotype:  Acute care utilization over past 12 months (defined as the number of admissions to the emergency department and/or acute care center as a result of pain crisis from chart review and biweekly telephone calls) after completing baseline pain assessment.  Chronic phenotype:  Subjects completed a baseline pain assessment using PAIN*Report*It during a routine outpatient visit (i.e., not during pain crisis or other urgent care visits to the clinic). A composite score (CPI) was computed as a representation of the multidimensional pain experience (location, intensity, quality, pattern) at baseline.  DNA extraction:  (1) blood samples: modified salting out procedure or QuickGene-mini80 isolation device & QuickGene DNA whole blood extraction method (2) buccal samples: modified phenol/chloroform procedure  Genotype:  MassARRAY iPlex Platform used for all SNPs except rs224534 and rs222747 (success rate >90%) | | Effect of *NR3CI* SNPs on acute (utilization) and chronic (CPI) pain phenotypes in SCD (IRR = incident rate ratio, B = unstandardized regression coefficient):   - rs33389   Utilization: IRR [95% CI]  Additive: 1.53 (1.09, 2.15) **p=0.014**  Recessive: 1.64 (1.12, 2.40) **p=0.011**   - rs2963155   Utilization: IRR [95% CI]  Additive: 1.80 (1.37, 2.38) **p=0.00003**  Recessive: 2.07 (1.45, 2.93) **p=0.00005**  Dominant: 2.25 (1.13, 4.49) **p=0.021**   - rs9324918   Utilization: IRR [95% CI]  Additive: 1.43 (1.06, 1.93) **p=0.021**  Recessive: 1.46 (1.00, 2.12) **p=0.050** | Findings from this exploratory study suggest evidence of an association between *NR3CI* (rs33389, rs2963155, rs9324918) and the heterogeneity of acute pain in SCD. |
| **(Powell-Roach et al., 2019)^7^**  107 individuals with SCD (Illinois, US)  Age (mean, SD) in years:  35.2 ± 12.0  range 19-70  Sex (n, %):  female 73 (68)  male 34 (32)  SC Type (n, %):  SCD-SS: 85 (79)  SCD-SC: 11 (10)  SCD-β^+^: 5 (5)  SCD-β^ο^: 5 (5)  SCD-α: 1 (1)  Race (n, %):  African American 104 (97)  Caucasian 3 (3)  Ethnicity (n, %):  Hispanic/Latina 2 (2)  Non-Hispanic 105 (98) | Acute  Chronic | Arginine Vasopressin Receptor 1A (*AVPR1A*)   - rs10877969 | | Acute phenotype:  Acute care utilization over past 12 months (defined as the number of admissions to the emergency department and/or acute care center as a result of pain crisis from chart review and biweekly telephone calls) after completing baseline pain assessment.  Chronic phenotype:  Subjects completed a baseline pain assessment using PAIN*Report*It during a routine outpatient visit (i.e., not during pain crisis or other urgent care visits to the clinic). A composite score (CPI) was computed as a representation of the multidimensional pain experience (location, intensity, quality, pattern) at baseline.  DNA extraction:  (1) blood samples: modified salting out procedure or QuickGene-mini80 isolation device & QuickGene DNA whole blood extraction method (2) buccal samples: modified phenol/chloroform procedure  Genotype:  MassARRAY iPlex Platform used for all SNPs except rs224534 and rs222747 (success rate >90%) | | Effect of *AVPR1A* SNP on acute pain (utilization) and stress as a pain aggravator:   - rs10877969   Utilization (mean, SD): **p=0.02**  Stress as a pain aggravator (%): **p=0.002**  Multivariate associations between genotypes and phenotypic variables (controlling for age, sex, sickle cell status):  Utilization (mean, SD): **p=0.01**  Stress as a pain aggravator: **p=0.003** | Findings from this exploratory study suggest evidence of an association between *AVPR1A* (rs10877969) and heterogeneity in acute pain and stress in SCD. |
| **(Sadhu et. al., 2018)^8^**  131 African American individuals with SCD (Illinois, US)  Age (mean, SD) in years:  34.3 ± 11.8  range 15-70  Sex (n, %):  female 86 (65.6)  male 45 (34.4)  SC Type (n, %):  SCD-SS: 102 (77.9)  SCD-SC: 15 (11.5)  SCD-β^+^: 7 (5.3)  SCD-β^ο^: 7 (5.3) | Acute  Chronic | GTP Cyclohydrolase 1 (*GCH1*)   - rs752688 - rs3783641 - rs4411417 - rs8007267 - rs10483639 | | Acute phenotype:  Acute care utilization over past 12 months (defined as the number of admissions to the emergency department and/or acute care center as a result of pain crisis from chart review and biweekly telephone calls) after completing baseline pain assessment.  Chronic phenotype:  Subjects completed a baseline pain assessment using PAIN*Report*It during a routine outpatient visit (i.e., not during pain crisis or other urgent care visits to the clinic). A composite score (CPI) was computed as a representation of the multidimensional pain experience (location, intensity, quality, pattern) at baseline.  DNA extraction:  (1) blood samples: modified salting out procedure or QuickGene-mini80 isolation device & QuickGene DNA whole blood extraction method (2) buccal samples: modified phenol/chloroform procedure  Genotype:  MassARRAY iPlex Platform used for all SNPs except rs224534 and rs222747 (success rate >90%) | | Effect of *GCH1* SNPs on acute (utilization) and chronic (CPI) pain phenotypes in SCD (IRR = incident rate ratio, B = unstandardized regression coefficient):   - rs3783641   Utilization: IRR [95% CI]  Additive: 1.37 (1.05, 1.81) **p=0.024**  Recessive: 1.81 (1.11, 3.05) **p=0.018**   - rs8007267   CPI: B (95% CI)  Additive: -3.76 (-7.28, -0.24) **p=0.037** Dominant: -5.61 (-10.37,- 0.85) **p=0.021** | Findings from this exploratory study suggest evidence of an association between *GCH1* rs3783641 with acute pain heterogeneity and *GCH1* rs8007267 with chronic pain heterogeneity. |
| **(Sadhu et. al., 2020)^9^**  131 individuals with SCD (Illinois, US)  Age (mean, SD) in years:  34.3 ± 11.8  range 15-70  Sex:  female 86 (65.6)  male 45 (34.4)  SC Type:  SCD-SS: 102 (77.9)  SCD-SC: 15 (11.5)  Others (SCD-β^+^,  SCD-β^ο^): 14 (10.7) | Acute  Chronic | Phenylethanolamine N-methyltransferase (*PNMT*)   - rs5638 - rs2934965 - rs876493 - rs2941523 | | Acute phenotype:  Acute care utilization over past 12 months (defined as the number of admissions to the emergency department and/or acute care center as a result of pain crisis from chart review and biweekly telephone calls) after completing baseline pain assessment.  Chronic phenotype:  Subjects completed a baseline pain assessment using PAIN*Report*It during a routine outpatient visit (i.e., not during pain crisis or other urgent care visits to the clinic). A composite score (CPI) was computed as a representation of the multidimensional pain experience (location, intensity, quality, pattern) at baseline.  DNA extraction:  (1) blood samples: modified salting out procedure or QuickGene-mini80 isolation device & QuickGene DNA whole blood extraction method (2) buccal samples: modified phenol/chloroform procedure  Genotype:  MassARRAY iPlex Platform used for all SNPs except rs224534 and rs222747 (success rate >90%) | | Effect of *PMNT* SNPs on acute (utilization) and chronic (CPI) pain phenotypes in SCD (IRR = incidence risk ratio, B = unstandardized regression coefficient):   - rs2934965   Utilization: IRR [97.5% CI]  Additive: 0.61 (0.38-1.01) **p=0.0.044**   - rs876493   Utilization: IRR [97.5% CI]  Additive: 0.68 (0.51-0.93) **p=0.012**  Dominant: 0.67 (0.47-0.97) **p=0.030**   - rs2941523   Utilization: IRR [97.5% CI]  Additive: 0.68 (0.47-0.99) **p=0.033**  Recessive: 0.12 (0.02-0.65) **p=0.017** | Findings from this exploratory study suggest evidence of an association between *PMNT* (rs2934965, rs876493, rs2941523) and heterogeneity of acute pain in SCD. |
| **(Kumar et al., 2021)^10^**  118 individuals with SCD from Jabalpur, India  Age (years):  median: 15  Sex (n, %):  Male 64 (54.2)  Female 54 (45.8) | Acute | KrÜppel-like factor 1 (*KLF1*)   - *KLF1*:c.-304 G > C - *KLF1*:c.-251 C > G   - rs3817621 - *KLF1*:c.304 T > C   - rs2072597 - *KLF1*:c.544 T   - rs2072596 - *KLF1*:c.*141 A > G - *KLF1*:c.*178 A > G | | Acute phenotype:  Pain episodes: (1) vaso-occlusive crises (VOC) characterized as any sickle cell disease-related crises including acute pain crisis which required hospitalization for pain management (2) pain episodes defined as pain in joints, bone, abdomen, or back ache that were not attributable to any other cause and did not require hospitalization for pain management.  DNA extraction:  Hemoglobin measured using Sysmex KX 21 and quantification of HbF and HbS using Bio-Rad  Genotype:  Not provided | | Effect of *KLF1* SNPs on number of hospital admissions per year:   - rs3817621   **p=0.015**  Effect of  *XMN-I* polymorphism  on number of pain episodes per year:  **p=0.004** | Findings from this exploratory study suggests evidence of an association between *KLF1* and the *XMN-I* polymorphism and acute pain heterogeneity in SCD. |
| **(Powell-Roach et al., 2022)^11^**  130 individuals with SCD (Illinois)  Age (mean, SD) in years:  35.0 ± 11.4  range 19-70  Sex (n, %):  Female 86 (66)  Male 44 (34)  SC Type (n, %):  SS 99 (76)  SC 15 (12)  Other 16 (12)  Ethnicity (n, %):  Hispanic 3 (2)  Non-Hispanic 128 (98) | Acute  Chronic | Catechol-O-Methyltransferase (*COMT*)   - rs2075507 - rs737865 - rs4646312 - rs4633 - rs6269 - rs165656 - rs165728 - rs165774 - rs174697 - rs740602 - rs769224 - rs4646310 - rs4646316 - rs9332377   Dopamine Receptor D3 (*DRD3*)   - rs167770 - rs167771 - rs2087017 - rs2399504 - rs3773679 - rs7611535 - rs1394016 - rs324023 - rs324026 - rs324029 - rs905568 - rs1800828 - rs2134655 - rs963468 - rs3732783 - rs9817063 | | Acute phenotype:  Acute care utilization over past 12 months (defined as the number of admissions to the emergency department and/or acute care center as a result of pain crisis from chart review and biweekly telephone calls) after completing baseline pain assessment.  Chronic phenotype:  Pain intensity measures were collected from routine outpatient visit using PAINReportIt, with average pain intensity (API) scores derived from mean of least and worst pain in the last 24 hours.  DNA extraction:  (1) blood samples: modified salting out procedure or QuickGene-mini80 isolation device & QuickGene DNA whole blood extraction method (2) buccal samples: modified phenol/chloroform procedure  Genotype:  MassARRAY iPlex Platform used for all SNPs except rs224534 and rs222747 (success rate >90%) | | Effect of *COMT* and *DRD3* haplotypes on utilization (acute) and API (chronic) pain phenotypes (p-value relative to reference haplotype):  *COMT* block 1:   - rs2075507 (A allele), rs4646310 (A allele), rs737865 (A allele)   Utilization **p=0.02**   - rs2075507 (A allele), rs4646310 (G allele), rs737865 (G allele)   API **p=0.02**  *DRD3* block 2:   - rs9817063 (T allele), rs2134655 (T allele), rs963468 (G allele), rs3773679 (C allele)   Utilization **p=0.01**  *DRD3* block 4:   - rs167770 (A allele), rs324029 (A allele), rs324023 (T allele)   Utilization **p=0.01**   - rs167770 (A allele), rs324029 (G allele), rs324023 (T allele)   Utilization **p=0.04**  API **p=<0.001** | Findings from this exploratory study suggest evidence of an association between haplotypes of *COMT* and *DRD3* and the heterogeneity of pain phenotypes in SCD. |
| **(Wang et al., 2022)^12^**  242 adults with HbSS at King’s College Hospital (KCH) in the United Kingdom (UK)  977 children with HbSS or HbSβ^ο^ from the Silent Infarct Transfusion Trial (SIT)  Age (mean, SD) in years:   - KCH adults   - 33.05 ± 11.26   - range 17.91-67.03 - SIT children   - 8.98 ± 2.43)   - range 5.03-14.99   Sex (n, %):   - KCH adults   - male 99 (41)   - female 143 (59) - SIT children   - male 513 (53)   - female 464 (47) | Acute | Pyruvate Kinase L/R (*PKLR*)  *KCH cohort*   - rs559809916 - rs1052177 - rs1052176 - rs138436282 - rs184056450 - rs143970593 - rs201306934 - rs116547266 - rs8177993 - rs8177991 - rs8177990 - rs139885057 - rs143289029 - rs4620533 - rs61208773 - rs8177982 - rs8177979 - rs8177977 - rs8177976 - rs2071053 - rs8177972 - rs8177970 - rs8177968 - rs8177967 - rs8177966 - rs116244351 - rs114455416 - rs78872377 - rs116186290 - rs114803660 - rs12067675 - rs144531972 - rs12741350 - rs147655495 - rs142347392 - rs530027982 - rs186226317 - rs11264357 - rs60856897 - rs3020781 - rs8177964 - rs8177963 - rs140638824 - rs531157617 - rs550843242 - rs74118436 | Pyruvate Kinase L/R (*PKLR*)  *SIT cohort*   - rs8847 - rs41364939 - rs932972 - rs8177994 - rs1052177 - rs1052176 - rs138436282 - rs184056450 - rs143970593 - rs187375238 - rs201306934 - rs116547266 - rs8177993 - rs8177991 - rs8177990 - rs182457669 - rs8177988 - rs3762272 - rs554407398 - rs184418772 - rs139885057 - rs143289029 - rs8177985 - rs4620533 - rs8177983 - rs528936663 - rs61208773 - rs8177982 - rs8177979 - rs8177977 - rs8177976 - rs115598568 - rs147689373 - rs541025103 - rs8177973 - rs200480300 - rs575392819 - rs2071053 - rs8177972 - rs201156800 - rs8177971 - rs538257138 - rs8177970 - rs138344553 - rs142339464 - rs550153962 - rs572844581 - rs8177968 - rs8177967 - rs8177966 - rs115736167 - rs8177965 - rs116244351 - rs112966658 - rs115499949 - rs114455416 - rs78872377 - rs116186290 - rs114803660 - rs12067675 - rs144531972 - rs12741350 - rs147655495 - rs142347392 - rs530027982 - rs565903017 - rs557773873 - rs186226317 - rs11264357 - rs549877553 - rs60856897 - rs3020781 - rs8177964 - rs8177963 - rs8177962 - rs8177961 - rs141571357 - rs183554608 | | Acute phenotype:  Acute sickle pain (defined as severe pain that could not be attributed to causes other than SCD and required hospitalization and treatment with opioid medication).  DNA extraction:  Not reported  Genotype:   - KCH adults   - Illumina Infinium “MEGA” chip - SIT children   - Illumina HumanHap650Y array 5 or Illumina Infinium HumanOmni 1-Quad array | Association of *PKLR* with annualized hospitalization rates in the KCH adult and SIT children cohorts:   - rs2071053   KCH adults: β -0.0883, **p=0.00097**  SIT children: β -0.0867, p=0.08140  Weighted Fisher’s meta-analysis combined **p=0.0009918**   - rs8177970   KCH adults: β 0.1299, **p=0.00036**  SIT children: β 0.0280, p=0.68660  Weighted Fisher’s meta-analysis combined **p=0.0042704**   - rs116244351   KCH adults: β 0.1247, **p=0.00064**  SIT children: β 0.0280, p=0.68660  Weighted Fisher’s meta-analysis combined **p=0.0068498**   - rs114455416   KCH adults: β 0.1247, **p=0.0064**  SIT children: β 0.0281, p=0.68600  Weighted Fisher’s meta-analysis combined **p=0.0068430**   - rs12741350   KCH adults: β -0.0864, **p=0.00115**  SIT children: β -0.0969**,** p=0.05160  Weighted Fisher’s meta-analysis combined **p=0.0007171**   - rs3020781   KCH adults: β -0.0864, **p=0.00115**  SIT children: β -0.0973, p=0.05080  Weighted Fisher’s meta-analysis combined **p=0.0007057**   - rs8177964   KCH adults: β 0.1241, **p=0.00071**  SIT children: β 0.0486, p=0.48950  Weighted Fisher’s meta-analysis combined **p=0.0050984** | Findings from this exploratory study suggest evidence of an association between *PKLR* ( rs2071053, rs8177970, rs116244351, rs114455416, rs12741350, rs3020781, rs8177964) and the heterogeneity of acute pain in adults with SCD. |
| **(Zhang et al., 2018)^13^**  438 individuals with SCD in walk-PHaSST study (9 US sites, 1 UK site)  Age (mean, SD) in years:  36.0 ± 12.5  Sex (n, %):  female 227 (51.8)  male 211 (48.2)  SC Type:  HbSS | Acute | Catechol-O-Methyltransferase (*COMT*)   - rs6269 - rs4633 - rs4818 - rs4680 - rs165599 | | Acute phenotype:  Health history survey with self-description of acute SCD pain, including number of episodes of acute pain in the last 30 days and one year prior to screening.  DNA extraction:  Qiagen kit  Genotype:  TaqMan platform and 7900 DNA analyzer | | Gender-specific effect of *COMT* SNPs on frequency of pain-related ER visits in the past month:  Rs4633   - all   **p=0.016**   - female   **p=0.007**  rs165599   - all   **p=0.009**   - female   **p=0.004** | Findings from this exploratory study suggest evidence of an association between *COMT* (rs4633, rs165599) and the heterogeneity of acute pain in SCD. |
| **(Galarneau et al., 2013)^14^**  387 US individuals with SCD from the Cooperative Study of Sickle Cell Disease (CCSD) replication cohort from 23 US Centers  Age (year):  11.2 ± 12.5  Sex (n, %):  female 210 (54.3)  male 177 (45.7)  318 individuals with SCD from Georgia Health Sciences University (GHSU) cohort  Sex (n, %):  female 169 (53.1)  male 149 (46.9)  Age (years):  Not reported  SC type:  Not reported  449 individuals with SCD from Duke cohort  Sex (n, %):  female 250 (55.7)  male 199 (44.3)  Age (year):  33.7 ± 12.1  SC type:  Not reported | Acute | Phospholipase A2 Group IVA (*PLA2G4A*)   - rs12720497   Uncharacterized gene (*LOC124907824*)   - rs540006   Interleukin 1 Receptor Type 1 (*IL1R1*)   - rs3917296   Dopamine D3 Receptor (*DRD3*)   - rs324035   Phospholipase C Eta 1 (*PLCH1*)   - rs10513478   Family With Sequence Similarity 193 Member A (*FAM193A*)   - rs11732673   TBC1 Domain Family Member 1 (*TBC1D1*)   - rs6858735   Melatonin Receptor 1A (*MTNR1A*)   - rs13113915   Adhesion G Protein-Coupled Receptor V1 (*ADGRV1*)   - rs10942625   Cytochrome P450 Family 3 Subfamily A Member 4 (*CYP3A4*)   - rs1851426   N-Acetyltransferase 1 (*NAT1*)   - rs10107231   Proprotein Convertase Subtilisin/Kexin Type 5  (*PCSK5*)   - rs7034457   Ribosomal Protein S24 (*RPS24*)   - rs7899453   Sortilin Related VPS10 Domain Containing Receptor 1 (*SORCS1*)   - rs11817401   Platelet Derived Growth Factor D (*PDGFD*)   - rs17101814   FTO Alpha-Ketoglutarate Dependent Dioxygenase (*FTO*)   - rs9933611   Ubiquitin conjugating enzyme E2 O (*UBE20*)   - rs445683   Ribonucleoprotein, PTB Binding 1 (*RAVER1*)   - rs7507634   Transketolase Like 1 (*TKTL1*)   - rs2872817 | | Acute phenotype:  Painful crisis (defined as an occurrence of pain lasting ≥ 2 hours in the extremities, back, abdomen, chest, or head not explained by a mechanism other than SCD).  DNA extraction:  Not reported  Genotype:  CSSCD and GHSU replication cohorts via MassARRAY iPlex platform. Duke cohort genotyped using the Illumina Human610-Quad SNP array. | | Effect of *FAM193A* SNPs on painful crisis:  CSSCD discovery   - rs11732673, β (SE): -0.338 (0.089), **p=2.2 x 10^-4^**   CSSCD replication   - rs11732673, β (SE): -0.374 (0.154), **p=0.02**   CSSCD combined   - rs11732673, β (SE): -0.347 (0.079), **p=9.9 x 10-6** | Investigators calculated an estimated 50% power to detect and association between the quantitative trait and SNP. Findings from this exploratory study suggest evidence of an association between *FAM193A* (rs11732673) and the heterogeneity of acute pain in SCD. |
| **(Al-Habboubi et al., 2012)^15^**  324 individuals with SCD (Bahrain, Middle East)  VOC group (n=210)  Age (mean, SD) in years:  11.3 ± 6.6  Sex (n, %):  male 121 (57.7)  female 89 (42.3)  SC type (mean, SD):  HbS 70.3 ± 9.9  Steady-state SCA controls (n=114)  Age (years):  13.5 ± 11.3  Sex (n, %):  male 62 (54.5)  female 52 45.5  SC type (mean, SD):  HbS 71.5 ± 9.4 | Acute | Vascular Endothelial Growth Factor A (*VEGFA*)   - rs699947 - rs833061 - rs1570360 - rs2010963 - rs833068 - rs833070 - rs3025020 - rs3025039 | | Acute phenotype:  Medical record review --VOC group included individuals with any vaso-occlusive crisis (VOC) event according to reported hospitalization, blood transfusion, and painful episodes in the last 9 months.  DNA extraction:  Not reported  Genotype:  VIC- and FAM-labeled primers and Assay-on-demand TaqMan assays | | Distribution of *VEGFA* genotype in SCD patients with VOC and SCD controls:   - rs2010963   **p=2.8 x 10^-6^**   - rs8333068   **p=3.5 x 10^-4^**   - rs3025020   **p=0.019**  Correlation of *VEGFA* SNPs and pain phenotypes:   - rs3025020   Hospitalization rates p = 0.010  Duration p = 0.038 | Averaging the power calculated from each SNP, investigators calculated an overall power of 82.4%. Findings from this study suggest evidence of an association between *VEGFA* (rs2010963, rs8333068, rs3025020) and the heterogeneity of acute pain in SCD. |
| **(Wonkam et al., 2018)^16^**  436 individuals with SCD (Cameroon, Africa)  Age (years):  median 16  range 5-54  Sex (n, %):  female 219 (50.3)  male 216 (49.7)  SC type:  Not reported | Acute | ATP Binding Cassette Subfamily B Member 1 (*ABCB1*)   - rs1045642   Adrenoceptor Alpha 1 (*ADRA1A*)   - rs1048101   Adrenoceptor Alpha 2A (*ADRA2A*)   - rs3750635   Adrenoceptor Beta 2 (*ADRB2*)   - rs1042713   Apolipoprotein L1 (*APOL1*)   - rs60910145 - rs73885319 - rs71785313   Arrestin Beta 2 (*ARRB2*)   - rs1045280   Arginine Vasopressin Receptor 1A (*AVPR1A*)   - rs10877969   BCL11 Transcription Factor A (*BCL11A*)   - rs11886868 - rs4671393   Bradykinin Receptor B2 (*BDKRB2*)   - rs1799722   Calcium Voltage-Gated Channel Auxiliary Subunit Alpha2delta 3 (*CACNA2D3*)   - rs1851048 - rs6777055   Catechol-O-Methyltransferase (*COMT*)   - rs4633 - rs6269 - rs4680   Dopamine Receptor D2 (*DRD2*)   - rs4274224   Fatty Acid Amide Hydrolase (*FAAH*)   - rs324419 - rs2295632 - rs4141964   HBS1 Like Translational GTPase- MYB Proto-Oncogene, Transcription Factor (*HBS1L-MYB*)   - rs28384513 - rs9376090 - rs9399137 - rs9389269 - rs9402686 - rs9494142   Hemoglobin Subunit Gamma 2 (*HBG2*)   - rs7482144   Potassium Voltage-Gated Channel Modifier Subfamily S Member 1 (*KCNS1*)   - rs734784   Opioid Receptor Mu 1 (*OPRM1*)   - rs1799971   Olfactory Receptor Family 51 Subfamily B Member 5 (*OR51B5/6*)   - rs5006884   Signal Transducer and Activator Of Transcription 6 (*STAT6*)   - rs841718 - rs3024971   Transient Receptor Potential Cation Channel Subfamily A Member 1 (*TRPA1*)   - rs920829   Transient Receptor Potential Cation Channel Subfamily V Member 1 (*TRPV1*)   - rs222747   UDP Glucuronosyltransferase Family 2 Member B7 (*UGT2B7*)   - rs7438135 | | Acute phenotype:  Medical record review for painful vaso-occlusive (VOC) events (defined as pain in the extremities, back, abdomen, chest or head that lasted at least two hours and not attributed to causes other than SCD, required a hospital visit, and treatment with non-opioid analgesics), consultation rates referring to outpatient visits, and hospitalization rates.  DNA extraction:  Puregene Blood Kit  Genotype:  TaqMan SNP Genotyping Assay and TaqMan Universal Master Mix and iPlex Gold Sequenom Mass Genotyping Array | | Variants in selected pain-related genes and VOC, consultation, and hospitalization rates:   - *CACNA2D3* (rs6777055):   VOC events  dominant model: **p=0.025**  Hospitalization rates  additive model: **p=0.008**   - *ADRB2* (rs1042713):   Consultation rates  recessive model: **p=0.0004**   - *UGT2B7* (rs7438135):   Consultation rates  additive model: **p=0.037**   - *COMT* (rs6269):   Hospitalization rates  dominant model: **p=0.027**   - *DRD2* (rs4274224):   VOC events  additive model: **p=0.037**   - *FAAH* (rs4141964):   Hospitalization rates  dominant model: **p=0.003**   - *KCNS1* (rs734784):   VOC events  over dominant model: **p=0.010**  Hospitalization rates  recessive model: **p=0.002**   - *BDKRB2* (rs1799971):   Hospitalization rates  over dominant model: **p=0.031**  Variants in known modifiers of sub-phenotypes of SCD, VOC, and hospitalization rates:   - *BCL11A* (rs11886868):   Hospitalization rates  additive model: **p=0.042**   - *BCL11A* (rs4671393):   VOC events  recessive model: **p=0.017**  Consultation rates  recessive model: **p=0.017**  Hospitalization rates  dominant model: **p=0.026**   - *HBSL1-MYB* (rs28384513):   Hospitalization rates  over dominant model: **p=0.010**   - *HBS1L-MYB* (rs9494142):   Hospitalization rates  dominant model: **p=0.038**   - *HGB2* (rs7482144):   Hospitalization rates  dominant model: **p=0.008** | Findings from this exploratory study suggest evidence of an association between *CACNA2D3* (rs6777055), *ADRB2* (rs1042713), *UGT2B7* (rs7438135), *COMT* (rs6269), *DRD2* (rs4274224), *FAAH* (rs4141964), *KCNS1* (rs734784), *BDKRB2* (rs1799971), *BCL11A* (rs11886868, rs4671393), *HBS1L-MYB* (rs28384513, rs9494142), and *HBG2* (rs7482144) and the heterogeneity of acute pain in SCD. |
| **(Rampersaud et al., 2021)^17^**  Jude’s Children’ Research Hospital Sickle Cell Clinical Research and Intervention Program (SCCRIP)  Age (mean, SD) in years:  5.8 ± 0.7  Sex (n, %):  male 168 (51.4)  female 159 (48.6)  SC type (n, %):  HbSS 316 (96.6)  HbSβ^0^-thalassemia 11 (3.4)  175 US and UK children with SCD from the Sleep and Asthma Cohort (SAC)  Age (mean, SD) in years:  10.8 ± 0.7  Sex (n, %):  male 92 (52.6)  female 83 (47.4)  SC type (n, %):  HbSS 165 (94.3)  HbSβ^0^-thalassemia 10 (5.7) | Acute | TNF Receptor Superfamily Member 1B (*TNFRSF1B*)   - rs1061622   Caspase 9 (*CASP9*)   - rs4645978   Fatty Acid Amide Hydrolase (*FAAH*)   - rs4141964 - rs324419 - rs2295632   Prostaglandin-Endoperoxide Synthase 2 (*PTGS2*)   - rs5275   Phospholipase A2 Group IVA (*PLA2G4A*)   - rs12720497   Interleukin 10 (*IL10*)   - rs3024498 - rs3024496 - rs1878672 - rs1518111 - rs1518110 - rs3024491   Interleukin 1 Receptor Type 2 (*IL1R2*)   - rs11674595   Interleukin 1 Alpha (*IL1A*)   - rs1800587   Interleukin 1 Beta (*IL1B*)   - rs1143634 - rs1143627   Interleukin 1 Receptor Antagonist (*IL1RN*)   - rs2234677   Potassium Inwardly Rectifying Channel Subfamily J Member 3 (*KCNJ3*)   - rs7574878 - rs2591168 - rs2591172   Sodium Voltage-Gated Channel Alpha Subunit 9 (*SCN9A*)   - rs6746030   Calcium Voltage-Gated Channel Auxiliary Subunit Alpha2delta 3 (*CACNA2D3*)   - rs1851048 - rs6777055   Dopamine Receptor D3 (*DRD3*)   - rs324035 - rs6280   Family With Sequence Similarity 193 Member A (*FAM193A*)   - rs11732673   TBC1 Domain Family Member 1 (*TBC1D1*)   - rs6858735   UDP Glucuronosyltransferase Family 2 Member B7 (*UGT2B7*)   - rs7438135   Melatonin Receptor Type 1A (*MTNR1A*)   - rs13113915   Adhesion G Protein-Coupled Receptor V1 (*GPR98*)   - rs10942625   Nuclear Receptor Subfamily 3 Group C Member 1 (*NR3C1*)   - rs2963155 - rs9324918   Adrenoceptor Beta 2 (*ADRB2*)   - rs1042713   Methionine Adenosyltransferase 2B-Teneurin Transmembrane Protein 2 (*MAT2B-TENM2*)   - rs7734804   Lymphotoxin Alpha (*LTA*)   - rs1799964   Tumor Necrosis Factor (*TNF*)   - rs1800629   Vascular Endothelial Growth Factor A (*VEGFA*)   - rs699947 - rs833061 - rs1570360 - rs2010963 - rs833068 - rs833070 - rs3025020 - rs3025039   Superoxide Dismutase 2 (*SOD2*)   - rs4880   Interleukin 6 (*IL6*)   - rs1800797 - rs1800796 - rs1800795   ATP Binding Cassette Subfamily B Member 1 (*ABCB1*)   - rs1045642   Cytochrome P450 Family 3 Subfamily A Member 4 (*CYP3A4*)   - rs1851426   Nitric Oxide Synthase 3 (*NOS3*)   - rs1800783   N-Acetyltransferase 1 (*NAT1*)   - rs10107231   GDNF Family Receptor Alpha 2 (*GFRA2*)   - rs17428041   Adrenoceptor Alpha 1A (*ADRA1A*)   - rs1048101   Transient Receptor Potential Cation Channel Subfamily A Member 1 (*TRPA1*)   - rs1947913 - rs13279503 - rs13255063 - rs1025928 - rs3735942 - rs3735943 - rs920829 - rs1443952   High Mobility Group Box 1 Pseudogene 46 (*HMGB1P46*)   - rs6986153   Potassium Two Pore Domain Channel Subfamily K Member 9 (*KCNK9*)   - rs3780039 - rs11166921   Proprotein Convertase Subtilisin/Kexin Type 5 (*PCSK5*)   - rs7034457   Mannose Binding Lectin 2 (*MBL2*)   - rs1800451 - rs7096206 - rs11003125   Ribosomal Protein S24 (*RPS24*)   - rs7899453   Sortilin Related VPS10 Domain Containing Receptor 1 (*SORCS1*)   - rs11817401   Hemoglobin Subunit Gamma 2 (*HBG2*)   - rs7482144   Matrix Metallopeptidase 1 (*MMP1*)   - rs1799750   Platelet Derived Growth Factor D (*PDGFD*)   - rs17101814   Dopamine Receptor D2 (*DRD2*)   - rs6277 - rs4274224   Signal Transducer and Activator of Transcription 6 (*STAT6*)   - rs841718   Arginine Vasopressin Receptor 1A (*AVPR1A*)   - rs10877969   Purinergic Receptor P2X 7 (*P2RX7*)   - rs208294 - rs7958311 - rs1718119   Protein Z (*PROZ*)   - rs3024718 - rs3024731 - rs3024735   NF-Kappa-B Inhibitor Alpha (*NFKBIA*)   - rs8904   GTP Cyclohydrolase 1 (*GCH1*)   - rs10483639 - rs7142517 - rs841 - rs752688 - rs4411417 - rs8007201 - rs7147286 - rs3783641 - rs3759664 - rs8007267   Galectin 3 (*LGALS3*)   - rs4644 - rs4652   Bradykinin Receptor B2 (*BDKRB2*)   - rs1799722   FTO Alpha-Ketoglutarate Dependent Dioxygenase (*FTO*)   - rs9933611   Transient Receptor Potential Cation Channel Subfamily V Member 1 (*TRPV1*)   - rs8065080 - rs224534 - rs222747   Arrestin Beta 2 (*ARRB2*)   - rs1045280   Protein Kinase C Alpha (*PRKCA*)   - rs887797   Ubiquitin Conjugating Enzyme E2 O (*UBE2O*)   - rs445683   Potassium Voltage-Gated Channel Modifier Subfamily S Member 1 (*KCNS1*)   - rs4499491 - rs734784 - rs6017486 - rs6073643   Interleukin 10 Receptor Subunit Beta (*IL10RB*)   - rs2834167   Potassium Inwardly Rectifying Channel Subfamily J Member 6 (*KCNJ6*)   - rs2835914 - rs8129919 - rs2836050   Catechol-O-Methyltransferase (*COMT*)   - rs6269 - rs4633 - rs4818 - rs4680 - rs165599   Calcium Voltage-Gated Channel Auxiliary Subunit Gamma 2 (*CACNG2*)   - rs4820242 - rs2284015 - rs2284017 | | Acute phenotype:  Acute vaso-occlusive pain (VOP) events (defined as visits to a health care facility that resulted in parenteral analgesics being administered).  DNA extraction:  QIAamp DNA Blood Mini Kit  Genotype:  Quant-iT dsDNA Assay Kit | | Associations of polygenic scores with VOP event rate:  VOP event rates:  _PGS_COMT **p=2.7 x 10^-5^**  _PGS_^5snps^ **p=5.8 X 10^-10^**  NOTE: 5 snps= sum of pain risk allele scores across *COMT* (rs6269, rs4633, rs4818, rs4680, rs165599)  Association of polygenic scores with VOP event occurrence:  VOP event occurrence:  PGS^COMT^ **p=2.0 x 10^-4^**  PGS^5snps^ **p=3.2 X 10^-9^**  NOTE: 5snps= sum of high pain risk alleles across 5 additional pain-related SNPs: *TBC1D1* (rs6858735),  *KCNJ6* (rs2835914),  *FAAH* (rs2295632), *NR3C1* (rs2963155), and *IL1A* (rs1800587) | Investigators calculated 94% and 48% power with a two-sided test to detect an association between SNPs and longitudinal VOP event rate in the SCCRIP cohort. For the SAC cohort, they calculated 95% power to detect an association. Investigators developed a polygenic score for acute vaso-occlusive pain. Findings from this study led to the identification of a 21-SNP, 9-locus PGS (*BCL11A, MYB, β-like globin gene cluster, COMT, TBC1D1, KCNJ6, FAAH, NR3CI, IL1A*) that provided evidence of a genetic association with acute pain heterogeneity in SCD. |
| **(Mahdi et al., 2012)^18^**  377 Bahraini Arabs with SCD  VOC group (n=239):  Age (mean, SD) in years:  14.2 ± 10.5  Gender (n, %):  male 141 (59)  female 98 (41)  SC type (mean, SD):  HbS 70.1 ± 12.7  Steady-state SCA group (n=138):  Age (mean, SD) in years:  11.9 ± 7.1  Gender (n, %):  male 75 (54.5)  female 63 (45.5)  SC type (mean, SD):  HbS 70.5 ± 11.6 | Acute | Protein Z (*PROZ*)   - rs3024718 - rs3024719 - rs3024731 - rs3024778 - rs3024772 - rs3024735 | | Acute phenotype:  Self-report of vaso-occlusive (VOC) painful episodes via interview of patients or guardians.  DNA extraction:  Not reported  Genotype:  TaqMan assays | | Correlation of *PROZ* genotypes with the following VOC parameters (ρ=rho coefficient, χ2=Pearson’s chi square):   - rs3024731   Type (generalized and localized): **ρ=0.156, χ2=1.5 x 10^-4^**  Age at onset (years): **ρ=0.136, χ2=0.001**  Duration of episode (days): **ρ=0.166, χ2=1.2 x 10^-4^**  Frequency (episodes/year): **ρ=0.174, χ2=1.8 x 10^-4^**  Affected site: **ρ=0.180, χ2=4.1 x 10^-5^**  Need for hospitalization: **ρ=0.165, χ2=8.0 x 10^-5^**  Pain scale (1-10): **ρ=0.148, χ2=0.001**  Treatment of pain: **ρ=0.161, χ2=1.1 x 10^-4^**   - rs3024735   Need for hospitalization: **ρ=0.139, χ2=0.001**  Pain scale (1-10): **ρ=0.096, χ2=0.021**  Treatment of pain: **ρ=0.109, χ2=0.005**  *PROZ* haplotype analysis of cases and controls (rs3024718, rs3024719, rs3024778, rs3024731, rs3024735, rs3024772) (χ2=Pearson’s chi square):   - AGGTG, **χ2=0.001** - GAAA, **χ2=0.024** - AGAA, **χ2=0.011** - GGTG, **χ2=0.002** | Investigators calculated an overall power of 79.05%. Findings from this study suggest evidence of a correlation between *PROZ* (rs3024731, rs3024735) and the heterogeneity of acute pain in SCD. Evidence from haplotype analysis suggests *PROZ* level deficiency may play a role in the pathogenesis of VOC. |
| **(Lettre et al., 2008)^19^**  1275 individuals with SCD from the African American Cooperative Study of Sickle Cell Disease (CSSCD):  Age (mean, SD) in years:  14.5 ± 12.1  Sex (n, %):  male 682 (53.5)  female 593 (46.5)  SC type:  Not reported  895 individuals from a subset of the CSSCD with SCD:  Age (mean, SD) in years:  16.9 ± 11.4  Sex (n, %):  male 484 (54.1)  female 411 (45.9)  SC type:  Not reported  350 Brazilians with SCD:  Age (mean, SD) in years:  11.4 ± 6.9  Sex (n, %):  male 162 (46.3)  female 188 (53.7)  SC type:  Not reported | Acute | BCL11 Transcription Factor A (*BCL11A*)   - rs4671393   HBS1 Like Translational GTPase- MYB Proto-Oncogene, Transcription Factor (*HBS1L-MYB*)   - rs28384513 - rs9399137 - rs4895441   β-globin gene cluster *XMN-I*   - rs7482144 | | Acute phenotype:  Painful crisis (defined as an occurrence of pain lasting ≥ 2 hours in the extremities, back, abdomen, chest, or head not explained by a mechanism other than SCD)  DNA extraction:  Not reported  Genotype:  Not reported | | Effect of  *HBS1L-MYB* on pain rate:   - *HBS1L-MYB* (rs4895441)   Estimated coefficient (SE, standard error): -0.306 (0.139), **p=0.028** | Findings from this exploratory study suggest evidence of an association between *HBS1L-MYB* (rs4895441) and the heterogeneity of acute pain in SCD. |
| **(Kalai et al., 2013)^20^**  200 individuals from Tunisia  SCA patients (n=100):  Age (mean, SD) in years:  30 ± 5  range 25-35  Sex (n, %):  male 36 (36)  female 64 (64)  SC type (mean, SD):  HbS 86.8 ± 0.7  Healthy controls (n=100):  Age (mean, SD) in years:  30 ± 5  range 25-35  Sex (n, %):  male 50 (50)  female 50 (50)  SC type:  None | Acute | C-C Motif Chemokine Ligand 5 (*CCL5*)   - rs2107538 - rs2280788 - rs2280789 | | Acute phenotype:  Painful crisis and infection  DNA extraction:  Phenol-chloroform standard procedure used to collect genomic data from peripheral blood sample  Genotype:  ABI PRISM Big Dye Termination ready reaction kit & ABI 310 DNA sequencer | | ----- | No significant findings for an association between *CCL5* SNPs and pain heterogeneity in SCD. |
| **(Belfer et al., 2014)^21^**  228 US SCD individuals from the NIH discovery cohort  Severe pain cases (n=155):  Sex (n, %):  male 78 (50.3)  female 77 (49.7)  Age (mean, SD) in years:  32.4 ± 10.0  SC type (n, %):  α-thalassemia 39 (32.8)  Controls (n=73):  Sex, (n, %):  male 31 (42.5)  female 42 (57.5)  Age (mean, SD) in years:  34.9 ± 13.6  SC type (n, %):  α-thalassemia 14 (31.8) | Acute | GTP Cyclohydrolase 1 (*GCH1*)   - rs8007267 - rs2878172 - rs3759664 - rs7147286 - rs841 - rs7142517 | | Acute phenotype:  In the NIH discovery cohort, cases were defined by ≥ 1 visit to an emergency department (or other acute care facility) or hospitalization for the treatment of acute sickle cell pain during the 12 months prior to evaluation as a patient reported outcome). In the CSSCD cohort, severe pain crisis was defined by any visit to a physician for treatment of sickle cell related pain lasting more than 2 hr.  DNA extraction:  Not reported  Genotype:  NIH cohort: TaqMan assays  CSSCD cohort: Illumina Human610-Quad SNP array | | Association with *GCH1* and severe pain crises in sickle cell anemia (NIH discovery and CSSCD replication cohort):   - rs8007267, major allele T   NIH discovery cohort:  dominant model OR (95% CI): 1.98 (1.11-3.53), **p=0.02**  CSSCD replication cohort:  dominant model OR (95% CI): 2.23 (1.29-3.84), **p=0.004**  rs2878172, major allele G  NIH discovery cohort:  dominant model OR (95% CI): 2.33 (1.23-4.41), **p=0.01**   - rs7147286, major allele A   NIH discovery cohort:  dominant model OR (95% CI): 2.13 (1.21-3.78), **p=0.009**  Unreplicated in CSSCD cohort | Findings from this exploratory study suggest evidence of an association between *GCH1* (rs8007267, rs2878172, rs7147286) and the heterogeneity of acute pain in SCD. |
| **(Bean et al., 2013)^22^**  820 Black or African American children (North America & Europe) from the Silent Infarct Transfusion Trial (SIT)  Age (mean, SD) in years:  8.9 ± 2.5  Sex (n, %):  male 429 (52.3)  female 391 (47.7)  SC type:  HbSS | Acute | *β-GLOBIN* cluster haplotype analysis using 5 SNPs   - rs11036351 - rs4320977 - rs16912210 - rs2855039 - rs7482144 | | Acute phenotype:  All vaso-occlusive events (VOE) that required hospitalization over a 3-year period prior to study enrollment were recorded and obtained via medical record review for each participant. Pain events were defined locally as episodes that could not be attributed to causes other than SCD and required hospitalization and treatment with opiates.  DNA extraction:  isolated from Epstein-Barr virus-transformed lymphoblast cells from participant blood  Genotype:  Illumina Infinium Human Omni1-Quad BeadChip, custom GoldenGate panel, or both | | ----- | No significant findings for an association between *β-GLOBIN* cluster haplotype and pain heterogeneity in SCD. |

References

1. Hu X, Jhun EH, Yao Y, et al. IL1A rs1800587 associates with chronic noncrisis pain in sickle cell disease. *Pharmacogenomics*. Dec 2016;17(18):1999-2006. doi:10.2217/pgs-2016-0085

2. Jhun E, He Y, Yao Y, Molokie RE, Wilkie DJ, Wang ZJ. Dopamine D3 receptor Ser9Gly and catechol-o-methyltransferase Val158Met polymorphisms and acute pain in sickle cell disease. *Anesth Analg*. Nov 2014;119(5):1201-7. doi:10.1213/ANE.0000000000000382

3. Jhun EH, Hu X, Sadhu N, et al. Transient receptor potential polymorphism and haplotype associate with crisis pain in sickle cell disease. *Pharmacogenomics*. Apr 2018;19(5):401-411. doi:10.2217/pgs-2017-0198

4. Jhun EH, Sadhu N, He Y, et al. S100B single nucleotide polymorphisms exhibit sex-specific associations with chronic pain in sickle cell disease in a largely African-American cohort. *PLoS One*. 2020;15(5):e0232721. doi:10.1371/journal.pone.0232721

5. Jhun EH, Sadhu N, Hu X, et al. Beta2-Adrenergic Receptor Polymorphisms and Haplotypes Associate With Chronic Pain in Sickle Cell Disease. *Front Pharmacol*. 2019;10:84. doi:10.3389/fphar.2019.00084

6. Jhun EH, Sadhu N, Yao Y, et al. Glucocorticoid receptor single nucleotide polymorphisms are associated with acute crisis pain in sickle cell disease. *Pharmacogenomics*. 08 01 2018;19(13):1003-1011. doi:10.2217/pgs-2018-0064

7. Powell-Roach KL, Yao Y, Jhun EH, et al. Vasopressin SNP pain factors and stress in sickle cell disease. *PLoS One*. 2019;14(11):e0224886. doi:10.1371/journal.pone.0224886

8. Sadhu N, Jhun EH, Yao Y, et al. Genetic variants of GCH1 associate with chronic and acute crisis pain in African Americans with sickle cell disease. *Exp Hematol*. 10 2018;66:42-49. doi:10.1016/j.exphem.2018.07.004

9. Sadhu N, Jhun EH, Posen A, et al. Phenylethanolamine N-methyltransferase gene polymorphisms associate with crisis pain in sickle cell disease patients. *Pharmacogenomics*. Mar 2020;21(4):269-278. doi:10.2217/pgs-2019-0096

10. Kumar R, Yadav R, Mishra S, et al. Kruppel-like factor 1 (KLF1) gene single nucleotide polymorphisms in sickle cell disease and its association with disease-related morbidities. Article. *Annals of Hematology*. Feb 2021;100(2):365-373. doi:10.1007/s00277-020-04381-z

11. Powell-Roach KL, Yao Y, Wallace MR, et al. Human study COMT and DRD3 haplotype-associated pain intensity and acute care utilization in adult sickle cell disease. *Exp Biol Med (Maywood)*. Mar 12 2022:15353702221080716. doi:10.1177/15353702221080716

12. Wang X, Gardner K, Tegegn MB, et al. Genetic variants of PKLR are associated with acute pain in sickle cell disease. *Blood Adv*. 2022;doi:10.1182/bloodadvances.2021006668

13. Zhang YZ, Belfer I, Nouraie M, et al. Association of genetic variation in COMT gene with pain related to sickle cell disease in patients from the walk-PHaSST study. *Journal of Pain Research*. 2018;11:537-543. doi:10.2147/jpr.S149958

14. Galarneau G, Coady S, Garrett ME, et al. Gene-centric association study of acute chest syndrome and painful crisis in sickle cell disease patients. *Blood*. Jul 18 2013;122(3):434-42. doi:10.1182/blood-2013-01-478776

15. Al-Habboubi HH, Mahdi N, Abu-Hijleh TM, Abu-Hijleh FM, Sater MS, Almawi WY. The relation of vascular endothelial growth factor (VEGF) gene polymorphisms on VEGF levels and the risk of vasoocclusive crisis in sickle cell disease. *Eur J Haematol*. Nov 2012;89(5):403-9. doi:10.1111/ejh.12003

16. Wonkam A, Mnika K, Ngo Bitoungui VJ, et al. Clinical and genetic factors are associated with pain and hospitalisation rates in sickle cell anaemia in Cameroon. *Br J Haematol*. Jan 2018;180(1):134-146. doi:10.1111/bjh.15011

17. Rampersaud E, Kang G, Palmer LE, et al. A polygenic score for acute vaso-occlusive pain in pediatric sickle cell disease. Article. *Blood Advances*. 2021;5(14):2839-2851. doi:10.1182/bloodadvances.2021004634

18. Mahdi N, Abu-Hijleh TM, Abu-Hijleh FM, Sater MS, Al-Ola K, Almawi WY. Protein Z polymorphisms associated with vaso-occlusive crisis in young sickle cell disease patients. Article. *Annals of Hematology*. 2012;91(8):1215-1220. doi:10.1007/s00277-012-1474-6

19. Lettre G, Sankaran VG, Bezerra MA, et al. DNA polymorphisms at the BCL11A, HBS1L-MYB, and beta-globin loci associate with fetal hemoglobin levels and pain crises in sickle cell disease. *Proc Natl Acad Sci U S A*. Aug 19 2008;105(33):11869-74. doi:10.1073/pnas.0804799105

20. Kalai M, Chaouch L, Ben Mansour I, Hafsia R, Ghanem A, Abbes S. Frequency of three polymorphisms of the CCL5 gene (rs2107538, rs2280788, rs2280789) and their implications for the phenotypic expression of sickle cell anemia in Tunisia. *Polish Journal of Pathology*. Jun 2013;64(2):84-89. doi:10.5114/pjp.2013.36012

21. Belfer I, Youngblood V, Darbari DS, et al. A GCH1 haplotype confers sex-specific susceptibility to pain crises and altered endothelial function in adults with sickle cell anemia. Article. *American Journal of Hematology*. 2014;89(2):187-193. doi:10.1002/ajh.23613

22. Bean CJ, Boulet SL, Yang G, et al. Acute chest syndrome is associated with single nucleotide polymorphism-defined beta globin cluster haplotype in children with sickle cell anaemia. Article. *British Journal of Haematology*. 2013;163(2):268-276. doi:10.1111/bjh.12507
