# Supplementary material for: Single nucleotide polymorphisms and sickle cell disease-related pain: a systematic review
Source: Front Pain Res (Lausanne). 2023 Sep 14;4:1223309. doi: 10.3389/fpain.2023.1223309 (PMC10538969; doi:10.3389/fpain.2023.1223309)
Supplement: Supplementary file 3 [file Datasheet3.docx]

**Supplemental Table 3.** Gene annotation for candidate-gene single-nucleotide polymorphisms (SNPs) associated with sickle cell disease pain phenotypes.

| SNP | Variant Type | Gene | Chr | Minor Allele | MAF | Functional Summary  (GeneCards/EntrezGene) | Gene Ontology Annotation |
| --- | --- | --- | --- | --- | --- | --- | --- |
| **Acute Pain Associated SNPs** | | | | | | | |
| RS17778257 | Upstream | *ADRB2* | 5q32 | T | 0.25 | Encodes beta-2-adrenergic receptor involved in mediation of catecholamine-induced activation of adenylate cyclase through the action of G proteins | G protein-coupled receptor activity; enzyme binding |
| RS12654778 | Upstream | *ADRB2* | 5q32 | A | 0.21 |  |  |
| RS11168070 | Upstream | *ADBR2* | 5q32 | G | 0.18 |  |  |
| RS11959427 | Upstream | *ADBR2* | 5q32 | C | 0.18 |  |  |
| RS1042711 | 5’ UTR | *ADBR2* | 5q32 | C | 0.20 |  |  |
| RS1801704 | 5’ UTR | *ADBR2* | 5q32 | C | 0.20 |  |  |
| RS1042713 | Missense | *ADBR2* | 5q32 | A | 0.50 |  |  |
| RS10877969 | Unspecified | *AVPR1A* | 12q14.2 | T* | 0.46 | Arginine vasopressin receptor: G-coupled protein receptor which mediates cell contraction & proliferation, platelet aggregation, release of coagulation factor & glycogenolysis | G protein-coupled receptor activity; protein kinase C binding |
| RS11886868  RS4671393 | Intron  Intron | *BCL11A*  *BCL11A* | 2p16.1  2p16.1 | T  G | 0.71  0.50 | Encodes a C2H2 type zinc-finger protein; among related pathways are signaling by ALK in cancer and infectious disease | Protein homodimerization activity; RNA polymerase II cis-regulatory region sequence-specific DNA binding |
| RS6777055 | Intron | *CACNA2D3* | 3p21.1-p14.3 | C | 0.20 | Mediates the influx of calcium ions into the cell upon membrane polarization | Voltage-gated calcium channel activity; calcium channel regulator activity |
| RS4680 | Missense | *COMT* | 22q11.21 | A | 0.31 | Catalyzes transfer of methyl groups responsible for major degradation pathway of catecholamine transmitters (dopamine, epi- and norepinephrine, thereby metabolizing endogenous substances & catecholamine drugs | Magnesium ion binding; catechol O-methyltransferase activity |
| RS4633 | Synonymous | *COMT* | 22q11.21 | T | 0.33 |  |  |
| RS165599 | Intron | *COMT* | 22q11.21 | A* | 0.31 |  |  |
| RS4680 | Missense | *DRD3* | 3q13.31 | A | 0.31 | Encodes D3 subtype of dopamine receptor which inhibit adenylyl cyclase and is found in the limbic areas of the brain associated with cognitive, emotional, and endocrine functions | G protein-coupled receptor activity; obsolete drug binding |
| RS4274224 | Intron | *DRD2* | 11q23.2 | A* | 0.31 | Encodes D2 subtype of dopamine receptor, which is involved in modulation of locomotion, reward, reinforcement, memory, learning | G-protein coupled receptor activity; identical protein binding |
| RS4141964 | Intron | *FAAH* | 1p33 | C* | 0.35 | Encodes a protein responsible for catalyzing hydrolysis of endogenous amidated lipids like the sleep-inducing lipid oleamide ((9Z)-octadecenamide), the endocannabinoid anandamide (N-(5Z,8Z,11Z,14Z-eicosatetraenoyl)-ethanolamine), as well as other fatty amides, to their corresponding fatty acids | Hydrolase activity; acting on carbon-nitrogen (not peptide) bonds, in linear amides, & carbon-nitrogen ligase activity with glutamine as amido-N-donor |
| RS11732673 | Intron | *FAM193A* | 4p16.3 | G | 0.17 | Encodes a protein and is a positive regulator p53 | Not available |
| RS3783641 | Intron | *GCH1* | 14q22.2 | A | 0.14 | Encodes a protein involved in first & rate-limiting enzyme in tetrahydrobiopterin (BH4) biosynthesis, an essential cofactor required by aromatic amino acid hydroxylases as well as nitric oxide synthases | Calcium ion binding; GTP binding |
| RS8007267^£^ | Unspecified | *GCH1* | 14q22.2 | C* | 0.40 |  |  |
| RS2878172 | Unspecified | *GCH1* | 14q22.2 | A* | 0.40 |  |  |
| RS7147286 | Intron | *GCH1* | 14q22.2 | G* | 0.34 |  |  |
| RS7482144 | Upstream | *HGB2* | 11p15.5 | A | 0.15 | Hemoglobin gamma G recombination region – may result in either deletions or expansions of the intervening sequence leading to hemoglobinopathies and/or thalassemias | Functional element |
| RS28384513  RS9494142 | Upstream  Downstream | *HBS1L-MYB*  *HBS1L-MYB* | 6q23.3  6q23.3 | G  C | 0.09  0.13 | Encodes a member of the GTP-binding elongation factor family expressed in multiple tissues with highest expression in heart and skeletal muscle; the intergenic region of this gene influences erythrocyte, platelet, and monocyte counts, erythrocyte volume, and hemoglobin content | GTP binding; translation elongation factor activity |
| RS734784 | Missense | *KCNS1* | 20q13.12 | T* | 0.44 | Encodes a voltage-gated potassium channel subunit which regulates resting membrane potential and the control of the shape and frequency of action potentials | Monoatomic ion channel activity; Delayed rectifier potassium channel activity |
| RS33389 | Intron | *NR3C1* | 5q31.3 | T | 0.10 | Encodes glucocorticoid receptor involved in inflammatory responses, cellular proliferation, & differentiation in target tissues | DNA-binding transcription factor activity; RNA polymerase II cis-regulatory region sequence specific DNA binding |
| RS2963155 | Intron | *NR3C1* | 5q31.3 | G | 0.27 |  |  |
| RS9324918 | Intron | *NR3C1* | 5q31.3 | C | 0.18 |  |  |
| RS1799971 | Missense | *OPRM1* | 6q25.2 | G | 0.04 | Encodes one of at least three opioid receptors in humans (mu); plays an important role in pain management & in drug dependence via modulation of dopamine system | G-protein coupled receptor activity; voltage-gated calcium channel activity |
| RS2071053 | Intron | *PKLR* | 1q22 | G | 0.38 | Encodes protein involved in the catalyzing of transphosphorylation of phosphoenolpyruvate into pyruvate and ATP, which is the rate-limiting step of glycolysis | Magnesium ion binding; pyruvate kinase activity |
| RS8177970 | Intron | *PKLR* | 1q22 | C | 0.09 |  |  |
| RS116244351 | Intron | *PKLR* | 1q22 | A | 0.12 |  |  |
| RS114455416 | Intron | *PKLR* | 1q22 | A | 0.12 |  |  |
| RS12741350 | Intron | *PKLR* | 1q22 | G | 0.00 |  |  |
| RS3020781 | Intron | *PKLR* | 1q22 | A* | 0.40 |  |  |
| RS8177964 | Intron | *PKLR* | 1q22 | A | 0.12 |  |  |
| RS2934965 | Upstream | *PMNT* | 17q12 | T | 0.07 | Catalyzes the last step of the catecholamine biosynthesis pathway, which methylates norepinephrine to form epinephrine (adrenaline) | Methyltransferase activity; phenylethanolamine N-methyl transferase activity |
| RS876493 | Intron | *PMNT* | 17q12 | A* | 0.30 |  |  |
| RS2941523 | Upstream | *PMNT* | 17q12 | G | 0.12 |  |  |
| RS3024731  RS3024735^+^ | Intron  Intron | *PROZ*  *PROZ* | 13q34  13q34 | A  A | 0.49  0.10 | Encodes a liver vitamin K-dependent glycoprotein that plays a role in regulating blood coagulation | Calcium ion binding; serine-type endopeptidase activity |
| RS33389 | Intron | *NR3C1* | 5q31.3 | T | 0.10 | Encodes glucocorticoid receptor involved in inflammatory responses, cellular proliferation, & differentiation in target tissues | DNA-binding transcription factor activity; RNA polymerase II cis-regulatory region sequence specific DNA binding |
| RS2963155 | Intron | *NR3C1* | 5q31.3 | G | 0.27 |  |  |
| RS9324918 | Intron | *NR3C1* | 5q31.3 | C | 0.18 |  |  |
| RS920829 | Missense | *TRPA1* | 8q21.11 | T | 0.27 | Central role in pain response to endogenous inflammatory mediators & various irritants (i.e., allythiocyanate, cinnamaldehyde, diallyl disulfide, acrolein); also, possibly pain response in cold perception, oxygen concentration perception | Monoatomic ion channel activity; channel activity |
| RS2010963 | 5’ UTR | *VEGF* | 6p21.1 | G* | 0.20 | Encodes a heparin-binding protein involved in proliferation and migration of vascular endothelial cells; required for physiological & pathological angiogenesis | Protein homodimerization activity; protein heterodimerization activity |
| RS833068 | Intron | *VEGF* | 6p21.1 | A | 0.47 |  |  |
| RS3025020 | Intron | *VEGF* | 6p21.1 | T | 0.09 |  |  |
| **Chronic Pain Associated SNPs** | | | | | | | |
| RS17778257 | Upstream | *ADRB2* | 5q32 | T | 0.25 | Encodes beta-2-adrenergic receptor involved in mediation of catecholamine-induced activation of adenylate cyclase through the action of G proteins | G protein-coupled receptor activity; enzyme binding |
| RS12654778 | Upstream | *ADRB2* | 5q32 | A | 0.21 |  |  |
| RS11168070 | Upstream | *ADBR2* | 5q32 | G | 0.18 |  |  |
| RS11959427 | Upstream | *ADBR2* | 5q32 | C | 0.18 |  |  |
| RS1042711 | 5’ UTR | *ADBR2* | 5q32 | C | 0.20 |  |  |
| RS1801704 | 5’ UTR | *ADBR2* | 5q32 | C | 0.20 |  |  |
| RS1042713 | Missense | *ADBR2* | 5q32 | A | 0.50 |  |  |
| RS8007267^£^ | Unspecified | *GCH1* | 14q22.2 | C* | 0.40 | Encodes a protein involved in first & rate-limiting enzyme in tetrahydrobiopterin (BH4) biosynthesis, an essential cofactor required by aromatic amino acid hydroxylases as well as nitric oxide synthases | Calcium ion binding; GTP binding |
| RS1800587 | Upstream | *IL1A* | 2q14.1 | A | 0.34 | Encodes interleukin cytokine involved in various immune responses, inflammatory processes, & hematopoiesis | Cytokine activity; interleukin-1 receptor binding |
| RS9722 | 3’ UTR | *SB100* | 21q22.3 | A | 0.39 | Involved in the regulation of cellular processes such as cell cycle progression & differentiation; may function in Neurite extension, proliferation of melanoma cells, stimulation of Ca2+ fluxes, inhibition of PKC-mediated phosphorylation, astrocytosis & axonal proliferation, & inhibition of microtubule assembly | Calcium ion binding; identical protein binding |
| RS1051169 | Synonymous | *SB100* | 21q22.3 | G* | 0.42 |  |  |
| RS9983698 | 3’ UTR | *SB100* | 21q22.3 | T | 0.18 |  |  |
| RS11911834 | Intron | *SB100* | 21q22.3 | T | 0.00 |  |  |

Note: MAF reported is for African American according to ALFA

*Minor allele in African American individuals is wild type allele in European individuals

+ G79A associated with RS3024735 in Mahdi article, but associated with Protein Z (PROZ) in NCBI

^£^ Associated with both Acute and Chronic Pain
